# Damping nonlinearity in agarose hydrogels under relative humidity: balancing network stiffness and energy dissipation

**DOI:** 10.1039/d6sm00405a

**Published:** 2026-07-17

**Authors:** Ikenna Ojoboh, Manuel Dedola, Katherine Nelms, Charles de Kergariou, Ibrahim Patrick, Ludovico Cademartiri, James P. K. Armstrong, Adam W. Perriman, Fabrizio Scarpa

**Affiliations:** a Bristol Composites Institute, University of Bristol Bristol BS8 1TR UK F.Scarpa@bristol.ac.uk; b Department of Chemistry, Life Sciences and Environmental Sustainability, University of Parma Parco Area delle Scienze 17 a Parma 43124 Italy; c Translational Health Sciences, Bristol Medical School, University of Bristol Bristol BS1 3NY UK; d School of Cellular and Molecular Medicine, University of Bristol Bristol BS8 1TD UK; e Research School of Chemistry and John Curtin School of Medical Research, Australian National University Canberra ACT 2601 Australia

## Abstract

Sustainable, biodegradable elastomers are needed to replace fossil-based alternatives and reduce the environmental impact of traditional vibration damping materials. We investigate agarose-based hydrogels as eco-friendly vibration absorbers, examining the combined effects of polymer concentration (1–7 wt%), relative humidity (55–98%), and mechanical pre-stress on their dynamic mechanical properties. Frequency-dependent viscoelastic and vibration transmissibility tests, supported by Gaussian process regression (GPR), reveal that increasing agarose concentration enhances the storage modulus (*E*′) by over an order of magnitude, reaching ∼5 MPa depending on humidity and applied prestress. Remarkably, the damping efficiency—characterised by the loss factor (tan(*δ*))—exhibits a highly non-monotonic trend. Maximum energy dissipation is observed at intermediate network densities, with tan(*δ*) up to 0.21 and a loss modulus of ∼515 kPa at 5 wt% and 75% relative humidity, comparable to synthetic elastomers and other advanced hydrogel composites. GPR analysis shows that prestress controls nonlinear stiffening and transmissibility resonance behavior, while shifting peak damping from 5 wt% to 1 wt% agarose as prestress increases. These findings underscore the mechanical tunability and sustainability of agarose hydrogels, providing potential design guidance for biodegradable vibration mitigation materials.

## Abstract 2

Sustainable, biodegradable elastomers are needed to replace fossil-based alternatives and reduce the environmental impact of traditional vibration damping materials.

## Graphical abstract

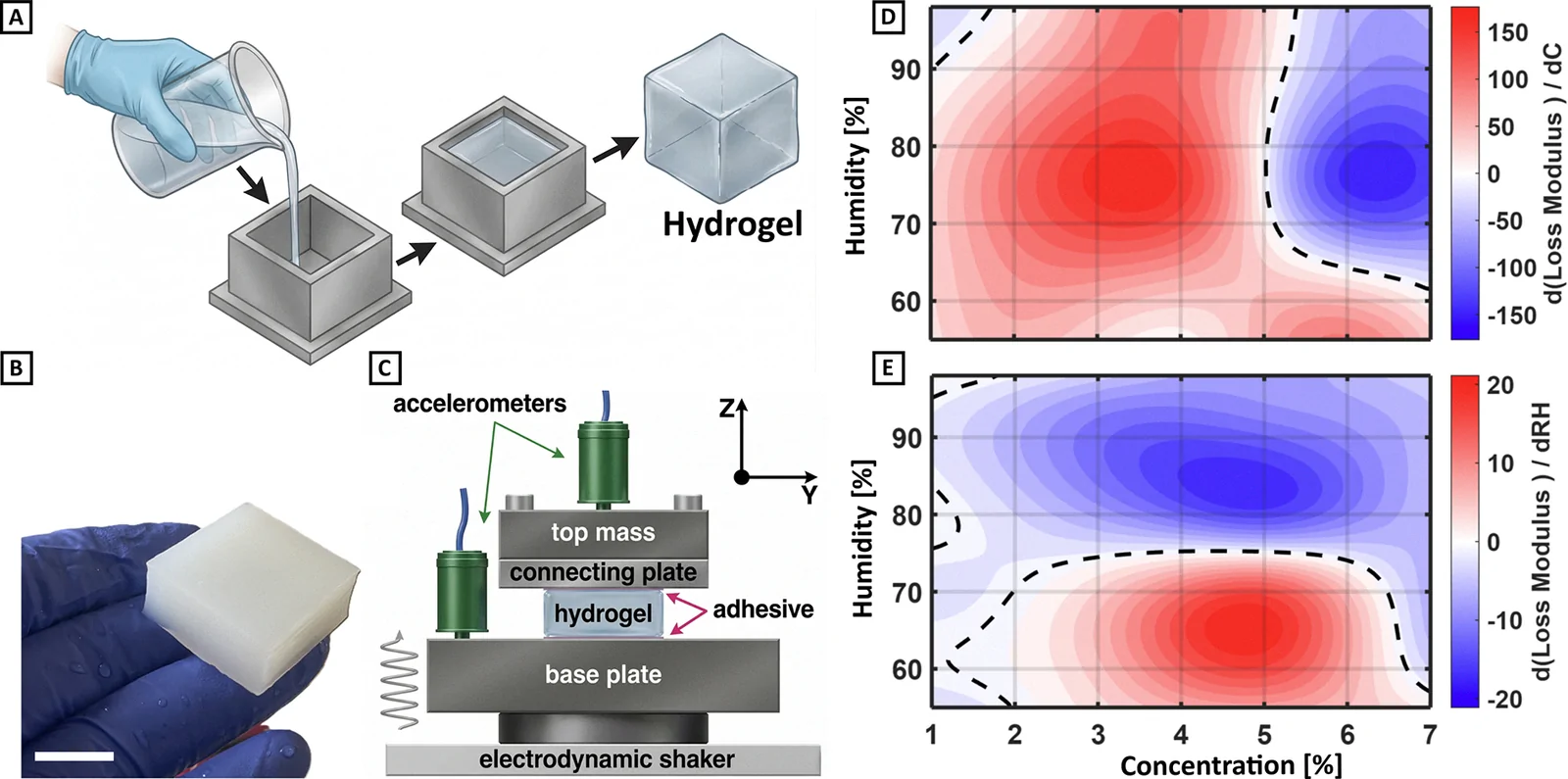

## 1 Introduction

The push for sustainable materials has accelerated the search for biodegradable alternatives to replace petroleum-based elastomers in vibration control.^1–3^ Conventional synthetic rubbers dissipate energy effectively *via* molecular viscoelasticity, but their environmental persistence drives demand for renewable soft materials with matching performance.^2^ In this context, hydrogels produced from biobased and biodegradable sources have recently emerged as highly promising candidates for vibration mitigation, offering highly tunable mechanical properties with significant viscous dissipation.^3,4^ The mechanical behavior of hydrogels stems from the balance between elastic energy storage and viscous dissipation driven by polymer chain mobility.^5^ In conventional single-network hydrogels, a higher polymer concentration generally increases stiffness but restricts molecular movement, which lowers viscous dissipation. Because of this, stiffness and damping are closely tied to the gel's structural network. This structure is highly sensitive to the exact polymer formulation^6^ and is strongly affected by thermal history and aging.^7,8^ However, stiffness and energy dissipation do not always follow a strict trade-off. Newer network designs, such as combining covalent and ionic crosslinks, show that these two properties can be successfully separated. This approach allows hydrogels to be both highly elastic and capable of massive energy dissipation.^9^

Despite promising vibration damping capabilities, hydrogels face key limitations. Hydrogels feature low stiffness, limited durability, sensitivity to temperature, humidity, with their properties significantly affected by mechanical prestress states. All these characteristics have restricted their adoption in mechanical engineering applications.^10,11^ Recent research has therefore focused on enhancing hydrogel performance through complex molecular and microstructure architectures, including double-network systems and composite formulations incorporating reinforcing fillers or fibres.^12–14^ Alginate–poloxamer systems exploit the thermoresponsive micellisation of poloxamer 407 to template network formation, yielding highly dissipative structures.^2,15^ Recent advances have demonstrated that doping these networks with natural reinforcements—such as sepiolite nanoclays and multiscale cactus fibers—can dramatically enhance dynamic stiffness and vibration damping, achieving loss factors exceeding 0.4 at frequencies below 300 Hz,^16^ a vibrational performance difficult to attain with classical fossil-based elastomers and polymer.^17^ The significant vibration damping behavior observed in the latter hydrogel composite systems also stems from slip-stick friction mechanisms existing between the natural fiber reinforcements and the matrix.^18,19^ While these approaches achieve significant damping, their structural complexity often obscures the underlying structure–property relationships existing the hydrogel system.^20^

**Fig. 1:**
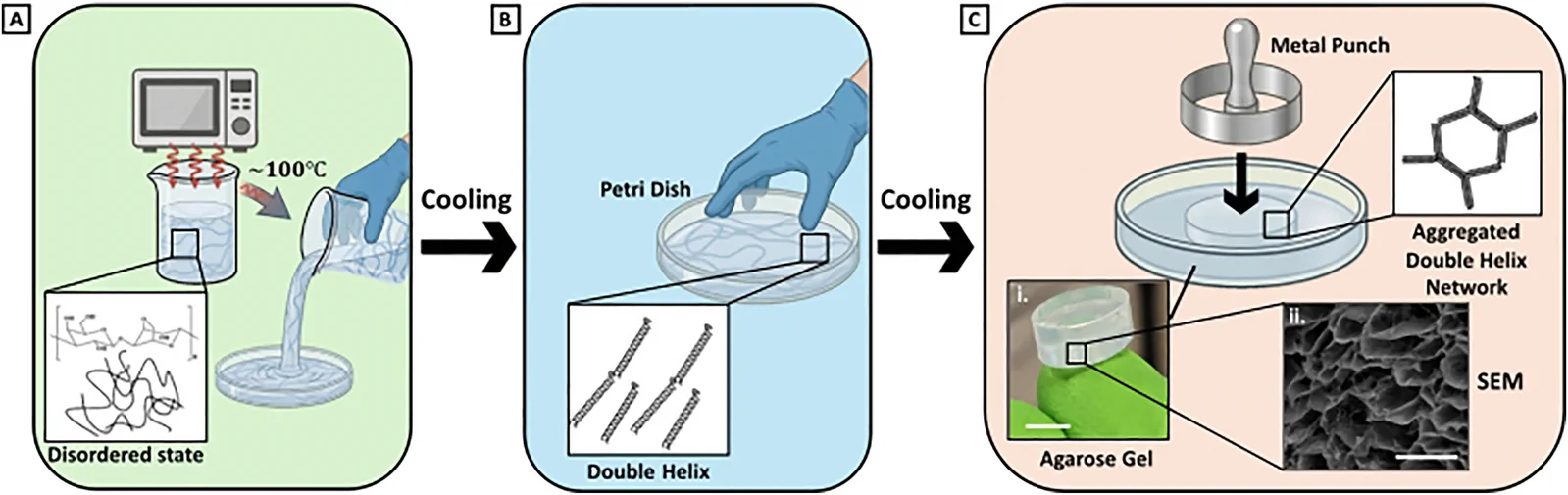
Structural evolution of agarose hydrogels during compression sample preparation. (A) Heating of agarose powder in aqueous solution to ∼100 °C *via* microwave irradiation, resulting in a disordered state. (B) Initial cooling phase within a Petri dish, where polymer chains transition into a double-helix conformation. (C) Formation of a stable agarose gel through further cooling and the development of an aggregated double-helix network. The final gel is processed using a metal punch for mechanical testing. Lyophilized agarose is characterized by scanning electron microscopy (SEM). The scale bar represents (i) 100 µm for SEM and (ii) 13 mm for the agarose gel at 1%.

Agarose is another class of hydrogel system which so far has been significantly used in the biomedical and tissue engineering sectors, but is promising candidate for damping applications. Agarose gels form thermoreversible networks, and their tunable gelation kinetics and adaptive mechanical properties drive their versatility as a controllable testing platform.^21,22^ Agarose solutions undergo a rapid sol–gel transition upon cooling, forming a 3D network dictated by their molecular structure and physicochemical properties. Gelation occurs in two stages (Fig. 1): initially, randomly distributed coils link *via* hydrogen bonds to form double-helical associations, followed by the aggregation (driven by hydrogen bond) of these helices into a rigid, three-dimensional network.^23,24^ The initial coil-to-helix transition that takes place during cooling can be described by a mean-field Zimm–Bragg approach.^25–27^ The architecture of agarose is controllable as well as less structurally complex than other composite hydrogels, making it an ideal platform for isolating viscoelastic damping mechanisms. Nonlinear, time-dependent behavior has been recently explored in other polysaccharide-based hydrogels, including mechanical memory (*i.e.*, history-dependent viscoelastic behavior and stress-softening phenomena)^28^ and cyclic structural rearrangements.^29^ However, the mechanisms underlying this behavior remain poorly understood, especially under realistic nonlinear loading conditions critical for advanced vibration mitigation.^30^ Moreover, key damping metrics, such as dynamic modulus (*E*′), loss modulus (*E*″), and loss factor (*η* or tan *δ*, are highly sensitive to subtle variations in concentration, hydration state and applied prestress, therefore necessitating systematic investigation.

In this study, we attempt to address these challenges through a comprehensive experimental and modeling framework to characterize biodegradable agarose hydrogels (1–7 wt%) under conditions relevant to practical vibration load applications. Vibration transmissibility testing under broad-spectrum white noise excitation is employed here to isolate the damping behavior. This approach enables direct quantification of frequency-dependent storage modulus (*E*′), loss modulus (*E*″), and loss factor (tan(*δ*)) across a wide parameter design space encompassing agarose concentration, relative humidity (55%–98%) and mechanical prestress. Our results show that while the storage modulus increases by over an order of magnitude with concentration—reaching up to ∼5 MPa, depending on environmental conditions and applied load—the damping efficiency follows a highly non-monotonic trend. Maximum energy dissipation is observed at intermediate network densities and hydration levels, where damping efficiencies reach values directly comparable to those of conventional synthetic elastomers and advanced hydrogel composites.^2,16,31^ Gaussian process regression (GPR) is also employed here to map the equivalent viscoelasticity measured using the vibration transmissibility tests across agarose concentrations, relative humidity and prestress values applied to the hydrogel samples. These predictive surfaces demonstrate that mechanical prestress primarily drives nonlinear stiffening and lowers resonance frequency, while also reshaping the damping landscape. As prestress increases, the optimal polymer concentration for maximum dissipation shifts from 5 wt% at low prestress to just 1 wt% at high prestress. This predictive framework may enable the identification of material and environmental design parameters that maximize energy dissipation in these biodegradable soft materials (Table 1).

**Table 1:** Mechanical and viscoelastic properties of agarose hydrogels. The fully hydrated state (98% RH) is compared to the damping state (75% RH) at a fixed top mass of *M* = 99 g

Concentration [%]	98% RH				75% RH		
	Young's modulus (*E*) [kPa]	*E*′ [kPa]	*E*″ [kPa]	tan *δ*	*E*′ [kPa]	*E*″ [kPa]	tan *δ*
1	47 ± 3	107	13	0.12	86	13	0.14
3	227 ± 135	923	44	0.05	1107	235	0.21
5	1076 ± 141	3118	243	0.08	2372	515	0.21
7	1339 ± 398	2734	173	0.06	3695	283	0.08

## 2 Materials and methods

### 2.1 Preparation of agarose hydrogels

#### 2.1.1 Synthesis

Agarose hydrogels with concentrations ranging from 1 to 7 wt% were prepared by dissolving related quantities of agarose BP160-100 powder (Fisher Scientific, USA) in deionized water. The solutions were autoclaved at 120 °C for 20 minutes to ensure complete dissolution, cooled to room temperature to allow initial gelation and subsequently refrigerated at 5 °C.

#### 2.1.2 Casting and shaping

Prior to sample fabrication, the gels were reheated to ∼100 °C until fully liquefied. The molten agarose was then cast into 3D-printed molds to form blocks of 30 × 30 × 15 mm (Fig. 3). Once solidified, the blocks were trimmed to provide a regular shape for the fitting in the vibration transmissibility rig (see SI, Tables S5–S7). Samples were also prepared for quasi-static compression testing. For that case, the liquefied agarose was poured into Petri dishes, allowed to gel, and cored using a metal punch to yield cylindrical specimens (Fig. 1C) to yield samples 13 mm of diameter and 5 mm of height. Due to the soft nature of the hydrogels, slight height variations occurred. To ensure accurate mechanical data normalization, the exact height (*L*_0_) of each specimen was measured with a caliper immediately before testing (see SI, Table S2).

#### 2.1.3 Conditioning of the specimens

Hydrogel samples were conditioned at three relative humidity (RH) values using monitored environmental conditions and solution or salts.^32^ The 55% RH was achieved in ambient controlled conditions, while the 75% and 98% with NaCl and K_2_SO_4_ solutions, respectively. Relative humidities below 25% were not investigated because the high water fraction of these gels (up to 99 wt% at 1 wt% agarose) leads to desiccation-driven collapse of the network and loss of viscoelastic function. Saturated salt solution were prepared with deionized water and excess salts, and stirred for 30 minutes to ensure saturation. The agarose hydrogels were placed within airtight plastic containers on platforms above the saturated salt solutions, sealed and refrigerated for 7 days to achieve the targeted RH conditions.^33^ Prior to testing, samples were removed from the conditioning chambers and tested immediately to prevent any environment-generated drift of their properties and performance. For both quasistatic compression and vibration transmissibility, the testing window was restricted to a few minutes to minimize exposure to ambient laboratory conditions.

### 2.2 Mechanical and dynamics characterization

#### 2.2.1 Quasistatic compression testing

Quasistatic uniaxial compression tests were performed using an FMS-500 L2 Force Measurement System (Starrett, Australia) equipped with a 100 N load cell. The top platen was lowered until initial contact with the sample surface was detected. During the compression test, a quasistatic displacement rate of 0.6 mm min^−1^ was applied. This rate corresponds to a strain of approximately ∼0.18% s^−1^ which falls below the critical threshold of 0.7% s^−1^ identified by Ed-Daoui *et al.*^34^ Operating within this range ensures a compressible poroelastic response, enabling interstitial fluid drainage during deformation.

#### 2.2.2 Vibration transmissibility testing

Vibration transmissibility tests were conducted using a dynamic shaker controlled *via* NI-DAQmx in MATLAB, interfaced with two data acquisition units (National Instruments USB-6211 and cDAQ-9178). The experimental apparatus consisted of a shaker powered by an LDS PA100E amplifier (gain = 5) and accelerometers (model 333 M07, PCB Piezotronics, USA) mounted on the rig's base and top plates (Fig. 3C).

Samples were centrally aligned, secured onto a rigid base plate, and prestressed by applying top masses (*M*) of 99 g, 146 g and 195 g. The masses were used to vary the resonant frequency of the transmissibility and therefore detect the variation of the dynamic/storage modulus and loss factor at those frequencies. The samples were subjected to white noise input at four acceleration amplitudes (0.1–0.625 m s^−2^) to assess responses linearity and characteristics. Input signals generated in MATLAB were transmitted using a NI USB-6211 DAQ and amplified through an LDS PA100E amplifier before being input to the shaker. Vibrations input to the base plate and output through the sample and were recorded using PCB accelerometers and a NI cDAQ-9178.^2^

#### 2.2.3 Dynamic mechanical analyzer

Longitudinal DMA was performed on a TA Instruments DMA850 equipped with a DMA-RH environmental control accessory. Frequency sweeps (0.1 to 100 Hz) were recorded at 55%, and 98% of relative humidity (RH). Tests were conducted in displacement control mode with an oscillation amplitude of 0.001 mm, corresponding to a dynamic strain of approximately ∼0.02%.

### 2.3 Data analysis and modeling

#### 2.3.1 Dynamic properties

Vibration response data were analyzed using MATLAB's tfestimate function within a custom-developed script to determine transfer functions correlating input and output signals. Dynamic mechanical properties, specifically the dynamic modulus *E*_d_ and loss factor *η*, were calculated from frequency response data from the following relationships:^2^1
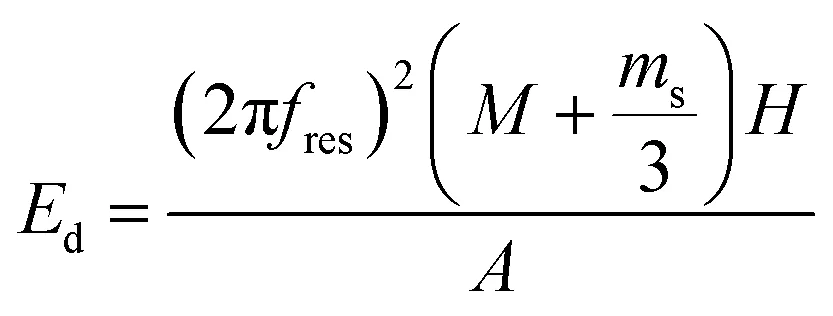


In (1), *f*_res_ is the resonance frequency, *m*_s_ is the hydrogel's mass, *M* is the top mass, *H* is the thickness and *A* is the cross-sectional area of the gel samples. The loss factor *η* was calculated as:2
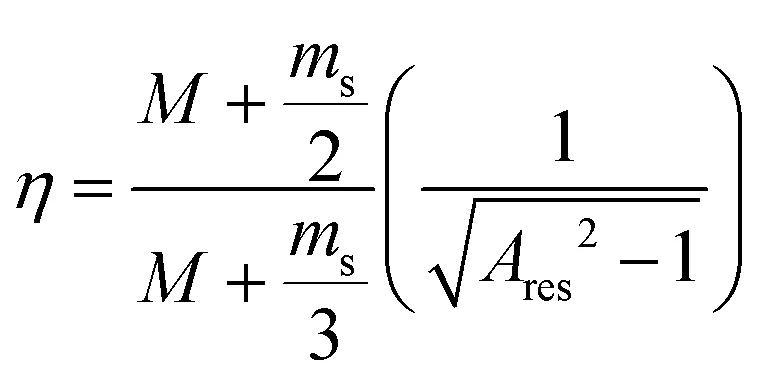


The term *A*_res_ in (2) represents the amplitude at the resonance frequency. The loss modulus was subsequently determined as the product of the dynamic modulus and the loss factor.^17^

#### 2.3.2 Compressive properties and hyperelastic modeling

The Young's modulus (*E*) was determined from the linear region of the stress–strain curve, specifically near the origin (*i.e.*, for strains < 0.05).^34,35^

To describe the large-strain behavior, the compression data were fitted to a two-parameter Mooney–Rivlin model of hyperelasticity.^36^ The relationship between the uniaxial stress (*σ*), the material parameters (*C*_01_ and *C*_10_), and the elongation (*λ*) is defined as:3
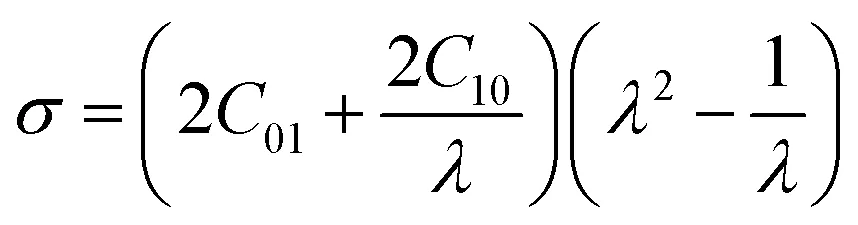


The material constants *C*_01_ and *C*_10_ were identified from the experimental quasi-static compression data *via* nonlinear least-square fitting into eqn (3).^36^

To quantify the energy absorption capacity within the compression range, a modulus of resilience *U*_*ε*_0__ was calculated as the area under the linear stress–strain (*σ*–*ε*) curve up to a fixed strain reference (*ε* = *ε*_0_):^37,38^4
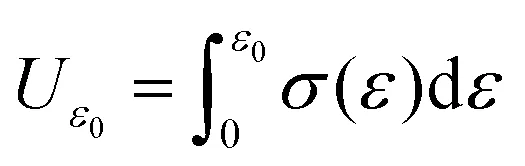


### 2.4 Gaussian process regression and model validation

#### 2.4.1 Model training and response surfaces

The multidimensional relationship between gel composition (agarose concentration), environmental conditions (relative humidity), loading (top mass), and the resulting viscoelastic properties (*E*′, *E*″, and tan *δ*) was modeled using Gaussian process regression (GPR) with automatic relevance determination (ARD) matérn 5/2 kernel and a constant basis function after previous standardization.^39,40^ Although other kernels provide strong numerical fits, the matérn 5/2 kernel was selected for its differentiable nature, which captures the smooth, continuous physical transitions intrinsic to the viscoelasticity of agarose gels. Additionally, a constant basis function was used to maintain a strictly non-parametric approach.

The final response surfaces and contour plots were generated by training the GPR models on the complete vibration transmissibility dataset.

#### 2.4.2 Grouped cross-validation procedure

A grouped *k*-fold cross-validation (*k* = 3) was performed to rigorously assess the predictive accuracy and applicability of the GPR framework while avoiding overfitting. The dataset was partitioned into three subsets using unique sample identifiers, ensuring all replicates of a given sample remained in the same fold. This “grouped” approach tests the model's ability to predict properties of entirely new gel formulations, rather than merely interpolating between identical replicates.^41^

#### 2.4.3 Performance metrics and reliability

The mathematical reliability of the generated surfaces has been quantified by the cross-validation coefficient of determination (*R*_CV_^2^). This metric is calculated as:^37^5
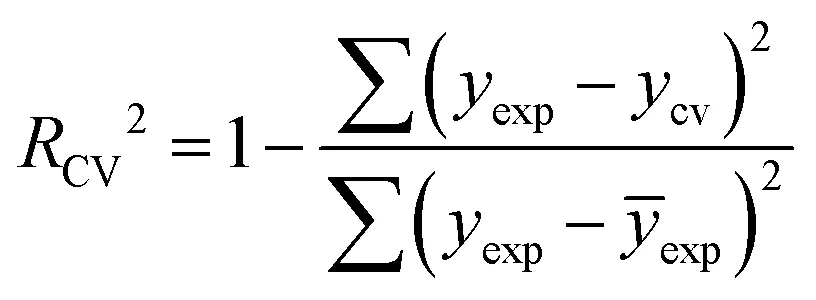
in (5), *y*_exp_ are the experimental values and *y*_cv_ are the “blind” predictions generated during the cross-validation cycles.

### 2.5 SEM images

Morphological analyses were performed using a FEI environmental scanning electron microscope (ESEM) equipped with a field emission gun (FEG) at the University of Parma. Images and spectra were recorded at an accelerating voltage of 7.0 kV under high vacuum.

### 2.6 Statistical analysis

Quasi-static compression tests employed five replicates (*n* = 5) to mitigate sensitivity to geometric defects and optimize hyperelastic model fitting.

A one-way analysis of variance (ANOVA), implemented using the {anova1}function, was conducted by considering the global *p*-value to assess the overall statistical significance of the data across all concentrations. DMA and vibration transmissibility tests were conducted using three replicates (*n* = 3) to minimize compositional variability arising from prolonged environmental exposure, thereby ensuring experimental consistency. As a consequence, dynamic measurements are reported here as mean values with their minimum and maximum limits. All mathematical modeling and statistical analyses were performed using MATLAB software.^42^

## 3 Results and discussion

### 3.1 Structural architecture and quasi-static compressive properties

The mechanical response of agarose hydrogels is fundamentally linked to their temperature-dependent sol–gel transition. Heating the agarose formulation in an aqueous solution to ∼100 °C induces a highly disordered state (Fig. 1A). Upon cooling to room temperature, these polymer chains undergo a conformational transition into double helices (Fig. 1B), which subsequently aggregate to form a structurally stable, three-dimensional porous network (Fig. 1C). Scanning electron microscopy (SEM) of the lyophilized samples (see SI, Fig. S1) confirms the presence of this architecture, revealing a highly interconnected, porous micro-structure capable of retaining substantial water volumes.

**Fig. 2:**
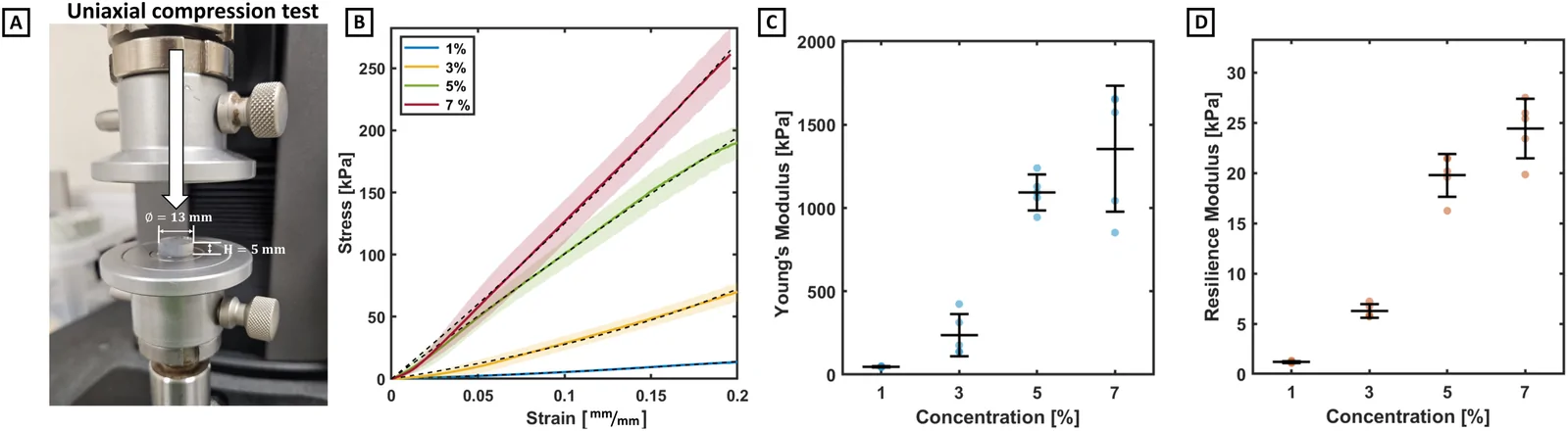
Compression and Mooney–Rivlin fitting of porous agarose hydrogels. (A) Schematic of the uniaxial compression test. The indicated height of 5 mm is a representative nominal value; actual specimen dimensions were recorded for each test to calculate stress and strain (see SI, Table S2). (B) Average stress–strain curves for hydrogels at varying agarose concentrations (1%, 3%, 5%, and 7%), obtained from *n* = 5 independent replicates. Solid lines represent the mean data, while shaded areas indicate the standard deviation. Dashed lines represent the Mooney–Rivlin fitting applied to the averaged experimental data. All fits show excellent agreement with the data (*R*^2^ > 0.99 for 1%, 3%, 5% and 7%). (C) Young's moduli from compression tests (compression speed 0.6 mm min^−1^). (D) Modulus of resilience calculated at *ε*_max_ = 0.20 for different agarose concentrations. Data in (C) and (D) are presented as individual data points (scatter) with mean horizontal lines; error bars denote the standard deviation. Statistical significance across groups in (C) and (D) was evaluated using a one-way ANOVA (global *p* < 0.01 indicates a significant effect of concentration).

The quasi-static uniaxial compression testing is considered here to be a baseline for assessing hydrogel properties. These were not conditioned prior to testing, but rather, were tested under ambient conditions immediately after casting and so were assumed to be fully hydrated (98% RH). Quasi-static uniaxial compression testing demonstrated a predictable, monotonic strain-stiffening effect associated with increasing polymer density. The Young's modulus, derived from the linear elastic region <5% strain, Fig. 2(A), increased by over an order of magnitude, scaling from 47 ± 3 kPa at 1 wt% concentration to 1339 ± 398 kPa at 7 wt% (Fig. 1C).^34,35^ This concentration-dependent stiffening is accompanied by a proportional increase in the modulus of resilience, rising from ∼1 kPa at 1 wt% to nearly 25 kPa at 7 wt%. This increase is an indication of the network's enhanced capacity for elastic energy storage under high-hydration states (Fig. 2D).

**Fig. 3:**
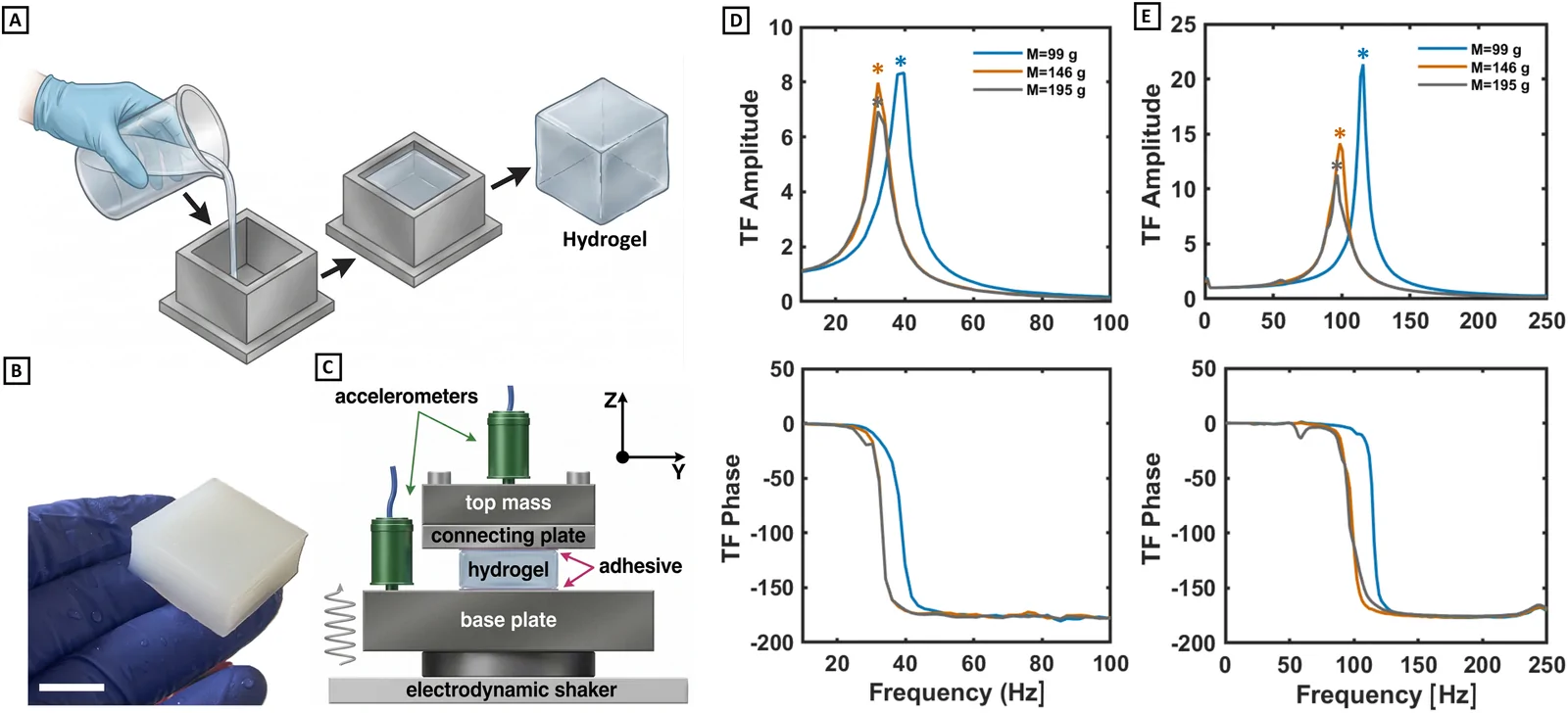
Vibrational transmissibility properties of agarose hydrogels at different concentrations. (A) Hydrogel block for vibration transmissibility experiments were prepared using a mold. (B) Optical image of a hydrogel block of dimensions 30 × 30 × 15 mm, scale bar demarcates 15 mm. (C) Schematic of the experimental setup. A hydrogel block is secured between a base plate and a connecting plate using alginate tray adhesive (magenta). To apply incremental compressive prestress, interchangeable top masses are secured to the connecting plate *via* screws. Accelerometers (green) mounted on the base plate and top mass monitor mechanical wave amplitudes before and after transmission through the hydrogel. (D) and (E) Vibration transmissibility data. Representative transfer function (TF) amplitude curves of agarose 1% and 3% (top row) with the corresponding TF phase curves (bottom row) at 98% of humidity. Asterisks indicate resonant frequency.

### 3.2 Hyperelastic behavior

Elastomeric and gel-like materials used in vibration damping are routinely subjected to large, non-linear strains and quasi-static forces.^17^ The experimental data from uniaxial compression were therefore further fitted to a two-parameters Mooney–Rivlin model that describe a baseline hyperelastic behavior. The experimental stress–strain curves shows good agreement with the model across all concentrations, with highly reliable fits (*R*^2^ > 0.99). The material parameters (*C*_10_ and *C*_01_) describe the uniaxial large-strains compression behavior of these agarose gels. By applying the relationship *E* = 6(*C*_10_ + *C*_01_), we also derived an expression for the theoretical Young's moduli (Table 2).

**Table 2:** Mooney–Rivlin parameters for agarose hydrogels. Comparison of *C*_01_ and *C*_10_ coefficients across different concentrations

Parameter [kPa]	Concentration [%]			
	1	3	5	7
*C*_01_	6.9	42.4	−163.5	−65.4
*C*_10_	0.6	−5.7	331.8	259.4
*E* = 6(*C*_10_ + *C*_01_)	45	220	1010	1164

The consistency between the theoretical predictions and the experimental data confirms the model's accuracy, with errors of 4.2%, 3.1%, 6.1%, and 13.1% across the 1–7% agarose concentration range. This close alignment confirms that the Mooney–Rivlin framework accurately describes the hyperelasticity and load-bearing capacity of the agarose matrix.

### 3.3 The influence of hydration

**Fig. 4:**
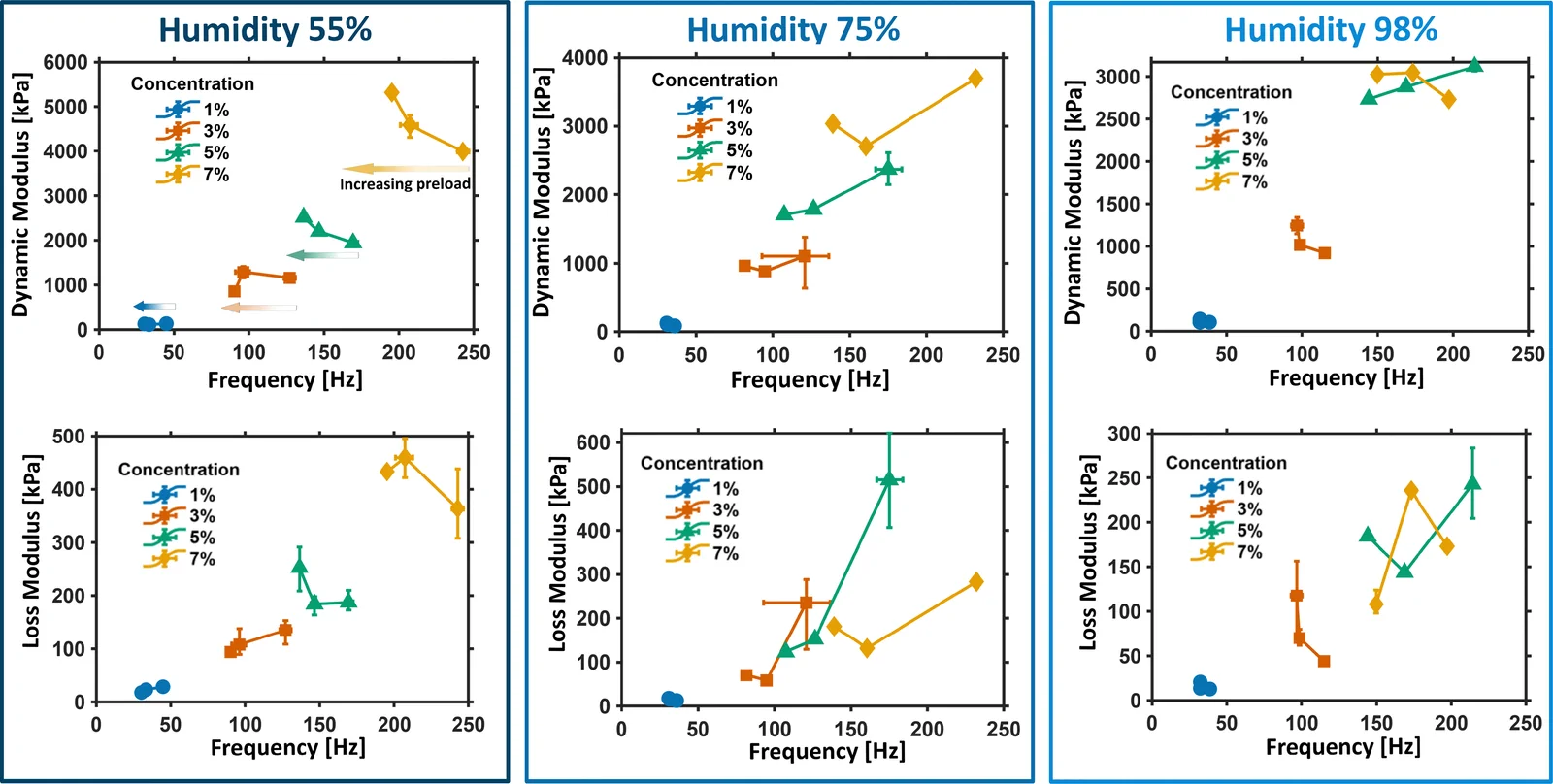
Vibrational transmissibility results for agarose hydrogels across a concentration range of 1–7% (wt%) and relative humidity levels (55, 75 and 98%). The storage modulus and the loss modulus are plotted as a function of resonance frequency. From left to right, the three columns represent increasing humidity levels. In each plot, for a given concentration curve, movement from right to left indicates increasing applied top mass (*i.e.*, rising prestress) respectively of 99 g, 146 g and 195 g. Error bars represent the minimum and maximum measured values for *n* = 3 independent samples.

Fig. 4 shows the relation between loss and the dynamic moduli at different values of humidity and prestress during vibrational transmissibility testing. While the fully hydrated state (98% RH) emphasizes the network’elastic storage capacity, effective vibration mitigation depends primarily on viscous energy dissipation. Damping efficiency depends on the balance between network stiffness and polymer chain friction. Our transmissibility tests conducted at 55%, 75%, and 98% RH show that this interplay is highly sensitive to environmental conditions: at full saturation (98% RH), the dynamic modulus (*E*′) reaches up to ∼3 MPa at 7 wt% concentration, but the water heavily lubricates the polymer chains, restricting viscous friction. Consequently, the loss factor (tan(*δ*)) is surprisingly low, dropping from 0.12 at 1 wt% to a minimal 0.06 at 7 wt%. Prior dynamic characterisation of agarose in low-strain, zero-prestress DMA conditions has reported higher loss contributions at low frequencies.^43^ The lower values observed here under vibration transmissibility loading likely reflect the combined effect of mechanical prestress and poroelastic fluid exudation under dynamic excitation, consistent with the minor fluid weeping observed during testing (see Fig. S7b and S8c).

To validate these trends, DMA compressive tests were conducted for 1.0 wt% agarose at 28 Hz and 98% humidity (see SI, Tables S10 and S11). This frequency was chosen to match the fundamental resonance of the 1.0 wt% system observed during vibration testing (Fig. 3D) while avoiding parasitic and inertial resonances in the vibration transmissibility setup. This cross-validation was restricted to the 1.0 wt% formulation as only these sampled had resonance modes within the frequency range possible using the DMA. However, we expect the trends across test methods observed for this formulation to hold for the other concentrations as well, given that the agarose concentration changes modulus quantitatively but not the underlying constitutive mechanisms.

The 1.0 wt% agarose exhibited a storage modulus of 32 ± 7 kPa under DMA compression. This value is close to the value of Young's modulus (47 ± 3 kPa) under quasistatic compression. The difference is likely attributed to the difference in magnitude of force applied, as the DMA measures moduli with only a few Newtons of force applied. That these two tests agree is supported by the vibration transmissibility tests for 1.0 wt% agarose under a 99 g top mass following conditioning at 98% humidity: this test yielded a significantly larger dynamic stiffness (*E*′ ≈ 107 kPa) in comparison to both the Young's modulus and storage modulus. At least in part, this discrepancy can be attributed to the prestress used during vibration testing. The tangent modulus from quasi-static compression curves at the corresponding applied stress of 7.3 kPa (corresponding to the 99 g top mass) yields a value of only 79.5 kPa for the 1.0 wt% gel (see SI, Table S3). Some weeping was observed during testing, especially for the lower concentration networks. Possibly, water expelled from the network under prestress further dehydrates the samples, as fluid weeping during testing was observed to increase for less dense networks. This water loss may give rise to a stiffening that cannot be captured by extrapolating the tangent modulus, though does support the above trend of stiffening as humidity is decreased. Moreover, the discrepancies between storage, Young's, and tangent moduli also raises the possibility of dynamic stiffening. Dynamic stiffening up to 7–10 times—where compressive properties vary with frequencies below 300 Hz during vibration transmissibility tests—has been previously reported in hydrogel systems such as alginate/poloxamer.^2,16^ It is expected here that vibration testing under white noise, high prestress, and relatively large displacement is much higher than what is possible with DMA at amplitudes on the order of microns and zero prestress. In short, the various moduli demonstrate the influence of prestress and dynamic stiffening on performance, and cross comparison accounting for the limits of each test method are in agreement with the vibrational transmissibility results. A comprehensive comparison of these modulus ratios across all concentrations at 98% RH is summarized in Table S3 of the SI.

**Fig. 5:**
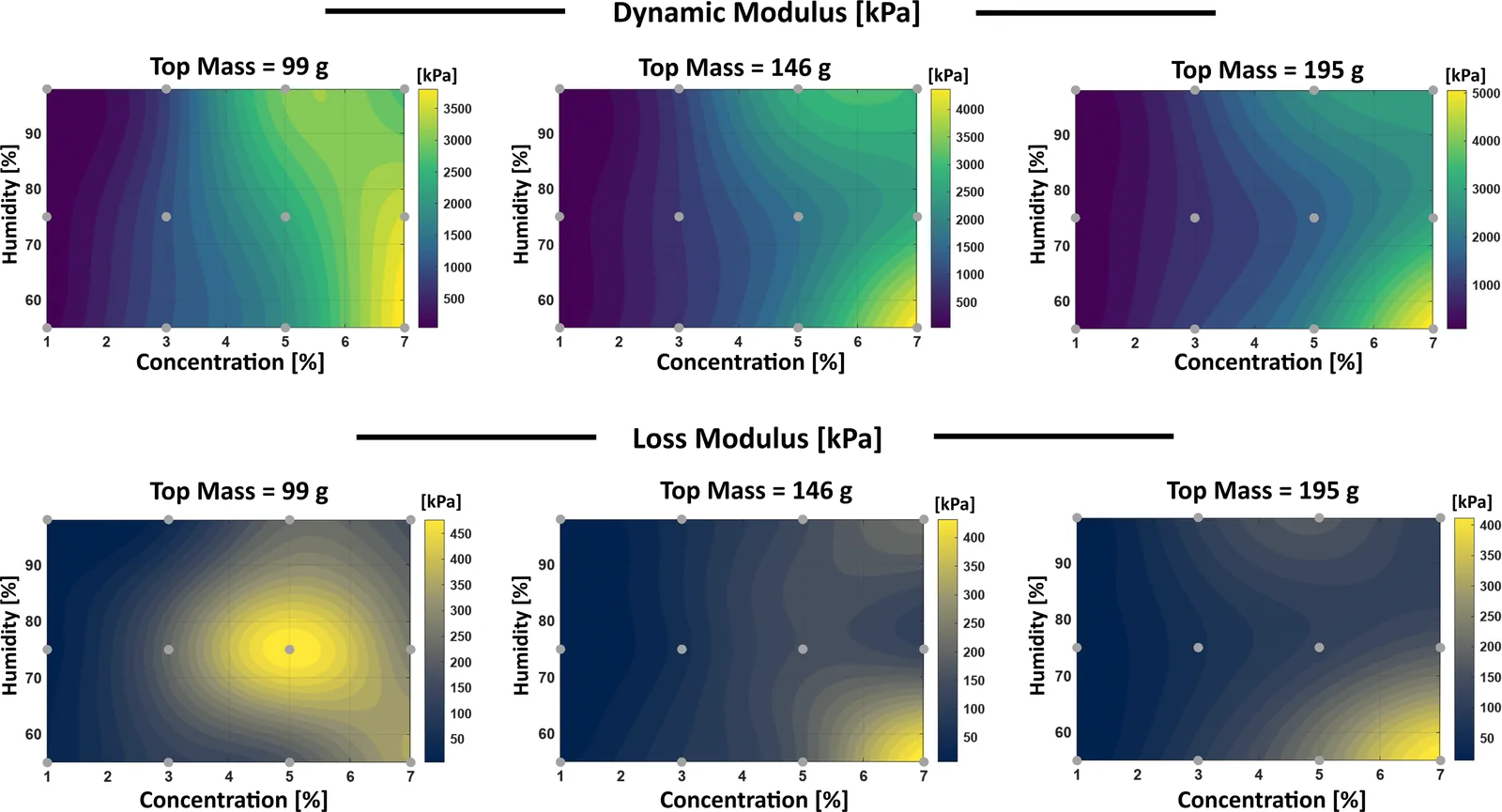
Gaussian process regression (GPR) predictive surfaces of hydrogel viscoelasticity. Contour maps illustrating the predicted dynamic modulus (top row, grouped CV RMSE: 122 kPa and grouped *R*_CV_^2^ = 0.99), and loss modulus (bottom row, grouped CV RMSE: 37 kPa and grouped CV *R*_CV_^2^ = 0.92) of agarose hydrogels as a continuous function of polymer concentration and relative humidity. The columns, from left to right, represent incrementally increasing compressive prestress (top mass) applied to the samples. Grey markers indicate the specific experimental data coordinates used to train the GPR model. The model uses an automatic relevance determination (ARD) matérn 5/2 kernel to capture the highly nonlinear, multidimensional dependency between mechanical damping and dynamic stiffness properties on hydration, network density and static pre-load.

Conditioning at an intermediate 75% RH produces a highly non-monotonic damping response during vibrational transmissibility testing, dramatically amplifying viscous dissipation (Fig. 5). The experiments showed peak energy dissipation at intermediate network densities, where tan(*δ*) reaches 0.21 and a corresponding loss modulus of 515 kPa at 175 Hz and 5 wt% concentration under 75% relative humidity. This damping performance exceeds the capacity of some standard commercial silicone rubbers (tan(*δ*) ∼ 0.12–0.18 at 15 Hz and room temperature).^44^ This damping performance also compares favorably with several conventional fossil-derived elastomers: it surpasses neoprene (tan *δ* 0.12–0.18^6^), is comparable to silicone rubber (tan *δ* 0.12–0.18 at 15 Hz^44^) and approaches the lower bound of butyl rubber (tan *δ* 0.2–0.4^6^). This is all achieved at a resonance frequency of 175 Hz and without the environmental persistence of fossil-derived materials. The values shown in this work are also highly comparable to the dynamic properties of recent advanced hydrogel architectures, such as alginate–poloxamer 407 systems (loss factors between 0.16 and 0.28 in the 100–300 Hz frequency range),^2^ alginate–sepiolite composite networks (loss factors exceeding 0.4 within the 80–140 Hz regime),^16^ and bioinspired hierarchical polyurethane–silicone composites (loss factor of 0.16 at a resonance frequency of 291 Hz).^31^

At this intermediate hydration level, internal chain friction is optimized while molecular rearrangement remains sufficiently active, resulting in damping efficiencies comparable to those of synthetic elastomers.^31,45^ However, dehydration to 55% RH significantly restricts chain mobility, resulting in network stiffening. The 7 wt% *E*′ exceeds 5 MPa under high prestress, while structural locking causes the previously optimal 5 wt% gel's *E*″ to decrease to ∼200 kPa. At 55% RH, only the densest 7 wt% network dissipates substantial energy (∼450 kPa), behaving predominantly as a glassy solid.

Although *in situ* characterization of the molecular network is beyond the scope of this study, these trends are conceptually interpreted as environmentally driven variations in network mobility. At 98% RH, water acts as a plasticizer, increasing chain mobility and suppressing damping efficiency. Conversely, at 55% RH, the network dehydrates and stiffens into a kinetically trapped state with reduced mobility. The dissipation peak observed at 75% RH reflects an optimal balance, whereby humidity-induced local ordering maximizes internal friction without causing excessive plasticization or network locking.^46^ Optimal vibration damping is therefore achieved by targeting the 75% RH regime, rather than simply increasing polymer concentration. Loss modulus values across the investigated prestress levels, concentrations and humidity conditions are reported in Table S7 of the SI.

### 3.4 Mapping the dynamic properties *via* predictive modeling

The Gaussian process regression effectively modeled the complex interactions observed in the vibration transmissibility test data by providing predictive surfaces (grouped cross-validated *R*_CV_^2^ = 0.99 for dynamic modulus and 0.92 for loss modulus; see Table S8 for the complete evaluation of GPR architectures). It is important to note that these predictive surfaces do not map the viscoelastic response at a single fixed frequency; rather, they represent the equivalent dynamic properties evaluated at the fundamental resonance frequency specific to each hydrogel configuration and applied preload. GPR predictive mapping, uncertainty analysis, and model validation for the different resonance frequencies are provided in Fig. S4, S5 and Table S9).

The GPR model is employed here as a tool to interpret trends within the experimentally sampled parameter space, rather than as a general predictive quantitative framework. Accordingly, extrapolations beyond the tested range should be avoided. The contour maps (Fig. 5) show that dynamic stiffness peaks at 7 wt% concentration and 55% RH, reaching up to 5 MPa under high preload. In contrast, the behavior of the energy absorbed by damping (*i.e.*, the equivalent loss modulus here) is more complex. Energy dissipation does not increase steadily. Instead, it peaks at 3–5 wt% concentration and 75% RH under low preload. The GPR model shows that damping changes with the load. As static preload increases, optimal damping surface peaks shift to lower polymer concentrations (1–3 wt%). This suggests that heavy static loads over-compress denser networks, thereby limiting internal chain friction. As a consequence, the optimal energy dissipation belongs to softer, less concentrated gel architectures. To assess the statistical reliability of these trends, the associated predictive uncertainty maps and 3D response surfaces are reported in Fig. S2 and S3, respectively. Expectedly, the predictive uncertainty increases away from the experimental sampling locations, and the increased predictive uncertainty at low concentrations reflects the wide range of moduli encompassed in the fitted GP model. With these in mind, the model serves to map the viscoelastic response strictly within the boundaries of our experimentally sampled parameter space, and the principal trends predicted are supported by the experimental data.

**Fig. 6:**
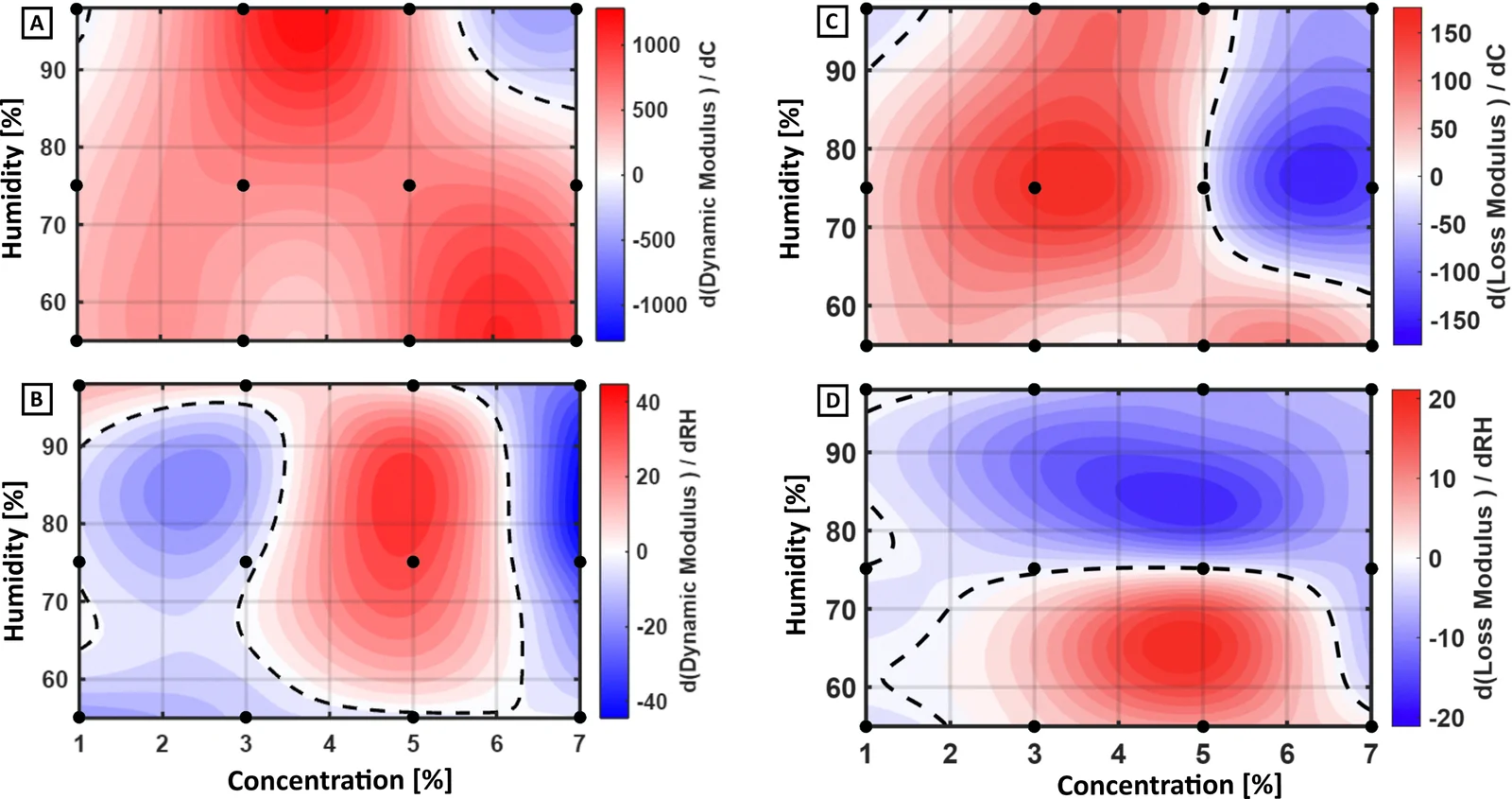
Viscoelastic sensitivity maps of agarose hydrogels evaluated at a constant preload mass of 99 g. The contour plots visualize partial derivatives of the material's dynamic response with respect to polymer concentration (top row: A, C) and relative humidity (bottom row: B, D). Columns correspond to the dynamic modulus (A) and (B) and loss modulus (C) and (D). Divergent color scales indicate the local rate of change, with the red and blue regions denoting positive and negative gradients, respectively. Dashed black lines represent zero-gradient contours, mathematically delineating critical structural and environmental transition zones where the material properties reach a local maximum, minimum, or plateau.

To obtain further insights into a more qualitative behavior of the dynamic properties of these agarose gels, the GPR surfaces were differentiated and partial derivatives were calculated numerically *via* the finite difference method using the MATLAB built-in gradient function to yield sensitivity maps at a constant 99 g of mass preload (Fig. 6).^42^ Viscoelastic sensitivity maps evaluated under the full range of prestress masses, from 99 g to 195 g, are reported in Fig. S6 of the SI. Analyzing partial derivatives with respect to polymer concentration (∂/∂*C*, top row) and relative humidity (∂/∂RH, bottom row) converts property maps into a stability analysis of the hydrogel networks under these dynamic conditions. Here, zero-gradient contours (dashed lines) define critical transition zones where damping efficiency remains robust against environmental changes. By contrast, steep positive (red) or negative (blue) gradients indicate regions where minor environmental changes induce rapid mechanical stiffening or a sudden collapse in internal chain friction.

The dynamic modulus reveals distinct stiffness sensitivities. Increasing polymer concentration globally stiffens the network (Fig. 6A), particularly at high levels of hydration. However, the sensitivity of the network's moisture response (Fig. 6B) is highly nonlinear. In leaner networks, increased humidity acts as a bulk lubricant, rapidly increasing compliance of the gel, as observed in Fig. 5. However, Fig. 6 shows that slight changes in humidity will affect stiffness by up to 1 MPa at high humidities for intermediate gel concentrations, and at low humidities for high concentrations. Overall, dynamic stiffness increases with concentration, though the susceptibility of the expected modulus initially decreases before increasing again at high humidities, and the sensitivity to humidity is much less than that on concentration. The trends in dynamic modulus and loss modulus as function of concentration are the same across the three top masses, though the sensitivity of dynamic modulus relative to humidity increases notably as the top mass is increased (Fig. S6 of the SI). These insights can inform damping applications, as they suggest what combinations of humidity and concentration are predicted to be least susceptible to environmental error.

The loss modulus maps define boundaries of optimal damping efficiency. Fig. 6C identifies a stability plateau at intermediate concentrations, bordered by a steep negative gradient (>5 wt%) where excessive polymer density rigidly locks the internal chains, decreasing damping capability. The figure also indicates the fundamental role of hydration at ∼75% RH, with a sharp transition in stability (Fig. 6D). This supports trends in loss modulus from Fig. 5, in which increased humidity causes increases before eventually a drop occurs, the drops being stronger for denser networks. In (Fig. 6D), the loss modulus is more susceptible to decrease as a function of increased humidity at high humidities, and more susceptible to increase with increased humidity below the transition point. Though the same trends in humidity are observed for the different prestresses, the stability landscapes show the opposite trend at high prestresses (Fig. S6 of the SI). The likely underlying mechanism is moisture liberates restricted chains, enhancing damping, until the point of overlubrication, causing damping efficiency to collapse. The onset of overlubrication is likely lower for greater prestress as the stress aids in overcoming inter-chain friction. These results confirm that while drier gels need moisture to enable inter-chain friction, oversaturation inhibits the polymer–polymer interactions essential for effective energy dissipation. It is worth noting, however, that the storage/dynamic modulus is only marginally affected by the level of humidity, with the wt% and the levels of pre-stress being the main factors affecting the performance of the agarose gels.

## Conclusions

This study reveals that the dynamic mechanical behavior of agarose hydrogels under varying environmental conditions is not solely determined by polymer concentration. Instead, it is critically influenced by the interplay of network density, environmental hydration, and mechanical prestress. While the hydrogels' network stiffness increases proportionally with the polymer fraction, damping exhibits a highly nonlinear dependency on environmental conditioning. We identified a structural optimal state at intermediate hydration (75% RH) and network density (3–5 wt%, with the loss-modulus maximum at 5 wt%), where the balance between polymer chain mobility and internal friction maximizes energy dissipation, yielding a loss factor (tan(*δ*)) of 0.21 at the resonance frequency of 175 Hz—performance directly comparable to conventional synthetic elastomers and advanced hydrogel composites.

Gaussian process regression (GPR) revealed that the mapping of the dynamic properties is load-dependent. Increased mechanical prestress reshapes the damping landscape, reducing overall loss modulus and shifting the maximum loss modulus for a given prestress to more dense networks. Further, the sensitivity of the different network architectures and environment is altered with loading conditions, shifting the conditions in which each architecture performs most effectively. These findings demonstrate that agarose hydrogels can be highly adaptive viscoelastic systems. By systematically mapping the influence of hydration and static preload on the internal hydrogels' network friction, this work establishes mechanistic design principles for engineering sustainable, high-performance soft materials tailored for vibration alleviation.

## Author contributions

Ikenna Ojoboh: methodology, investigation, formal analysis, writing – original draft. Manuel Dedola: investigation, formal analysis, data curation, validation, writing – original draft. Katherine Nelms: methodology, supervision, validation, project administration, writing – review and editing. Charles de Kergariou: methodology, formal analysis, data curation, resources. Ibrahim Patrick: methodology, supervision. Ludovico Cademartiri: supervision, formal analysis. James P. K. Armstrong: supervision, resources. Adam W. Perriman: conceptualization, funding acquisition. Fabrizio Scarpa: conceptualization, supervision, resources, project administration, funding acquisition, writing – review and editing.

## Conflicts of interest

There are no conflicts to declare.

## Supplementary Material

SM-022-D6SM00405A-s001

## Data Availability

The data supporting the findings of this study are available within the article and its supplementary information (SI). Supplementary information: all experimental data relevant to the manuscript's preparation and formulation of theories and/or hypotheses, including conclusions *etc.* See DOI: https://doi.org/10.1039/d6sm00405a. Raw data are available from the corresponding author upon reasonable request.
