# Supplementary material for: Damping nonlinearity in agarose hydrogels under relative humidity: balancing network stiffness and energy dissipation
Source: Soft Matter. 2026 Jul 17;22(33):5571–81. doi: 10.1039/d6sm00405a (PMC13454995; doi:10.1039/d6sm00405a)
Supplement: Supplementary file 1 [file SM-022-D6SM00405A-s001.pdf]

Supplementary Information (SI) for Soft Matter.
This journal is © The Royal Society of Chemistry 2026

Damping nonlinearity in agarose hydrogels under relative humidity: balancing network stiffness and energy dissipation

SUPPORTING INFORMATION

Ikenna Ojoboh[a], **Manuel Dedola**[b], **Katherine Nelms**[a], **Charles de Kergariou**[a], **Ibrahim Patrick**[a], **Ludovico Cademartiri**[b], **James P. K. Armstrong**[c], **Adam W. Perriman**[d,e] and **Fabrizio Scarpa**[*b]

[a] Bristol Composites Institute, University of Bristol, Bristol, BS8 1TR, United Kingdom.
[b] Department of Chemistry, Life Sciences and Environmental Sustainability, University of Parma, Parco Area delle Scienze 17 a, Parma 43124, Italy.
[c] Translational Health Sciences, Bristol Medical School, University of Bristol, Bristol BS1 3NY, United Kingdom.
[d] School of Cellular and Molecular Medicine, University of Bristol, Bristol BS8 1TD, United Kingdom.
[e] Research School of Chemistry and John Curtin School of Medical Research, Australian National University, Canberra, ACT 2601, Australia.

* *Corresponding author: F.Scarpa@bristol.ac.uk*

S1 SEM OF AGAROSE SAMPLES

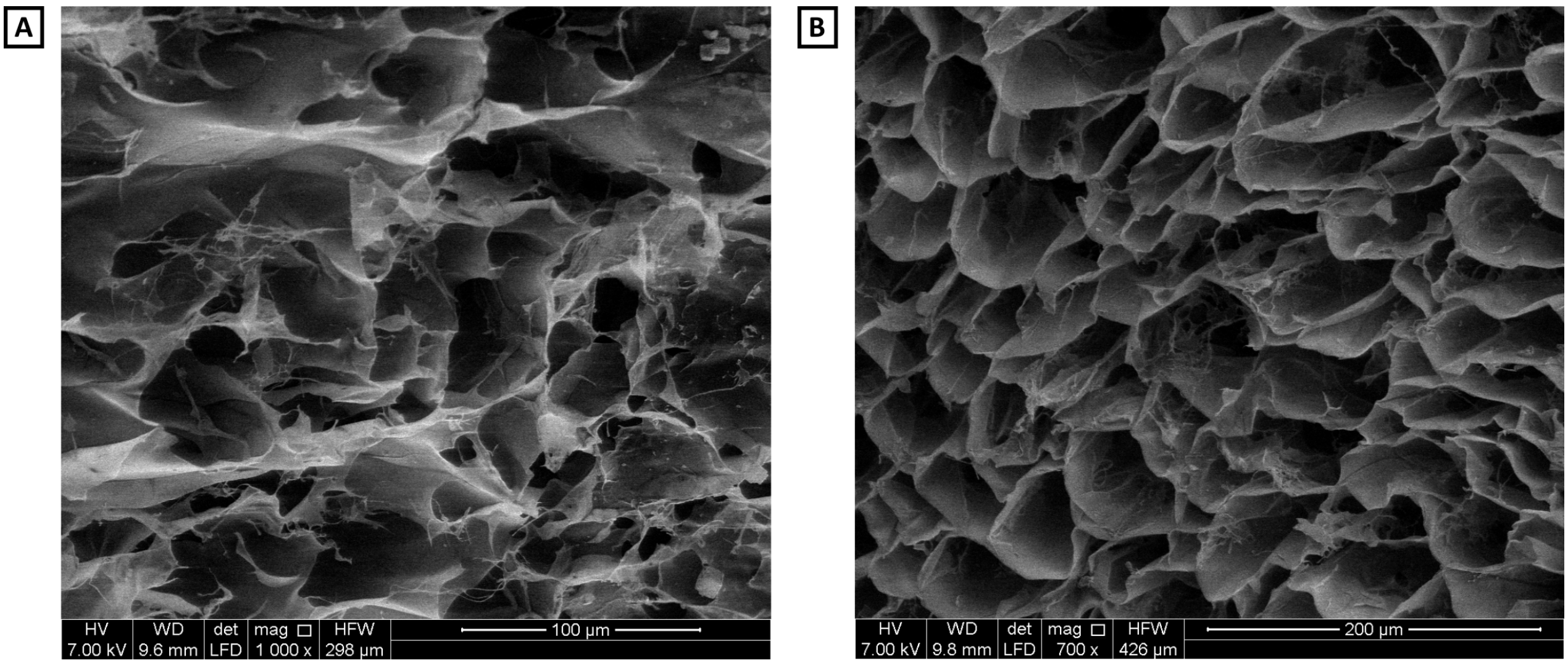

Figure S1: Scanning electron micrographs (SEM) of lyophilized 1 wt% agarose. **(A)** Surface morphology. **(B)** Internal cross-section of the sample.

S2 SUMMARY OF EXPERIMENTAL MODULUS COMPARISONS

Table S1: Summary of modulus ratios at 98% RH ($E(x\%)/E(1\%)$, comparing DMA storage modulus (E'), Young's modulus, tangent modulus (E_{tan}), and vibration transmissibility dynamic modulus (E') under preload mass of 195 g.

**Conc. [%]**	**DMA E' Ratio (98% RH)**	**Young's Modulus Ratio (98% RH)**	**E_{tan} Ratio (98% RH, 195 g)**	**Vibr. Trans. E' Ratio (98% RH, 195 g)**
1%	1.00	1.00	1.00	1.00
3%	3.50	4.83	4.87	8.74
5%	3.41	22.89	11.80	19.20
7%	7.84	28.49	14.98	21.22

S3 AGAROSE SAMPLE DATA: QUASISTATIC COMPRESSION TESTS

Table S2: Thickness [mm] used in Quasistatic compression (compression speed 0.6 mm/min).

Conc.	Sample	Diameter [mm]	Thickness [mm]
		**1% Agarose**	
1%	1	13.0	5.0
1%	2	13.0	5.0
1%	3	13.0	4.5
1%	4	13.0	4.7
1%	5	13.0	5.7
		**3% Agarose**	
3%	1	13.0	5.0
3%	2	13.0	5.0
3%	3	13.0	5.0
3%	4	13.0	5.0
3%	5	13.0	5.0
		**5% Agarose**	
5%	1	13.0	5.0
5%	2	13.0	6.0
5%	3	13.0	6.0
5%	4	13.0	6.0
5%	5	13.0	6.0
		**7% Agarose**	
7%	1	13.0	5.0
7%	2	13.0	5.0
7%	3	13.0	5.0
7%	4	13.0	5.0
7%	5	13.0	5.0

Table S3: Tangent modulus under varying pre-stress conditions, calculated from mean stress-strain curves.

Conc.	Mass [kg]	Stress [kPa]	Strain [mm/mm]	Tangent Modulus [kPa]
1%	0.099	7.3	0.124	79
1%	0.146	10.8	0.164	89
1%	0.195	14.4	0.211	82
3%	0.099	7.3	0.041	288
3%	0.146	10.8	0.052	334
3%	0.195	14.4	0.062	399
5%	0.099	7.3	0.010	806
5%	0.146	10.8	0.014	897
5%	0.195	14.4	0.018	968
7%	0.099	7.3	0.012	924
7%	0.146	10.8	0.015	1138
7%	0.195	14.4	0.018	1228

S4 AGAROSE SAMPLES DATA: VIBRATION TRANSMISSIBILITY TESTING

Table S4: Agarose Samples data at 20°C and 55% RH.

Conc.	Sample	Thickness [mm]	Length [mm]	Breadth [mm]	Mass [g]	Top Mass [g]
			**1% Agarose**			
1%	1	15.3	29.3	29.0	14.0	195
1%	2	14.5	29.0	29.0	13.0	146
1%	3	14.1	29.1	29.0	13.2	99
			**3% Agarose**			
3%	1	14.7	29.0	28.8	13.7	99
3%	2	15.0	29.0	29.2	13.3	146
3%	3	15.1	29.0	29.2	13.7	195
			**5% Agarose**			
5%	1	14.4	29.4	29.5	13.2	99
5%	2	14.9	29.4	29.4	13.5	146
5%	3	15.0	29.4	29.7	13.8	195
			**7% Agarose**			
7%	1	15.3	29.5	29.5	13.8	146
7%	2	15.5	29.5	29.7	14.0	195
7%	3	14.7	29.7	29.8	13.0	99

Table S5: Agarose Samples data at 20.7°C, Sample RH = 75%.

Conc.	Sample	Length [mm]	Breadth [mm]	Thickness [mm]	Mass [g]	Top Mass [g]
			**1% Agarose**			
1%	1	29.0	28.2	13.4	11.9	99
1%	2	28.2	28.5	14.5	11.8	146
1%	3	29.4	29.8	14.4	13.6	195
			**3% Agarose**			
3%	1	26.3	26.0	12.7	9.2	195
3%	2	29.5	29.7	14.5	14.3	146
3%	3	29.0	29.3	15.3	15.3	99
			**5% Agarose**			
5%	1	27.7	27.5	14.5	11.4	99
5%	2	27.8	27.6	14.5	11.7	146
5%	3	27.3	27.8	14.3	11.4	195
			**7% Agarose**			
7%	1	29.7	29.6	14.7	15.1	99
7%	2	26.6	26.1	13.9	10.7	195
7%	3	29.5	29.6	15.4	14.3	146

Table S6: Agarose Samples data at 20.2°C, Sample RH = 98%.

Conc.	Sample	Length [mm]	Breadth [mm]	Thickness [mm]	Mass [g]	Top Mass [g]
			**1% Agarose**			
1%	1	29.0	28.7	14.7	13.3	99
1%	2	29.0	29.1	14.6	13.4	146
1%	3	29.1	28.8	14.6	13.3	195
			**3% Agarose**			
3%	1	29.5	29.6	14.9	13.5	99
3%	2	29.2	29.4	15.1	13.6	146
3%	3	29.5	29.5	14.7	13.3	195
			**5% Agarose**			
5%	1	29.7	29.6	14.6	13.7	99
5%	2	29.7	29.7	15.0	13.8	146
5%	3	29.7	29.6	14.7	13.7	195
			**7% Agarose**			
7%	1	29.2	29.2	14.7	12.9	99
7%	2	29.1	29.3	14.6	12.9	146
7%	3	29.4	29.4	14.8	13.1	195

Table S7: Mean Loss Modulus [kPa] of agarose hydrogels as a function of concentration and relative humidity for different top masses. Data are presented as Mean (Min - Max) for $n = 3$.

Top Mass [g]	Conc. [%]	RH 55%	RH 75%	RH 98%
99	1	29 (22 - 43)	13 (12 - 13)	13 (13 – 13)
	3	135 (116 - 162)	235 (125 - 443)	44 (42 - 46)
	5	187 (173 - 211)	515 (407 - 621)	243 (205 - 284)
	7	364 (211 - 568)	283 (251 - 309)	173 (17 - 179)
146	1	23 (19 - 29)	15.8 (15 - 16)	14 (13 - 14)
	3	94 (91 - 97)	58 (55 - 61)	70 (68 - 71)
	5	184 (165 - 198)	152 (146 - 161)	144 (144 - 146)
	7	459 (420 - 489)	132 (131 - 133)	236 (226 - 242)
195	1	18 (14 - 20)	18 (17 - 18)	18 (17 - 19)
	3	108 (107 - 110)	70 (69 - 71)	118.4 (111 - 123)
	5	253 (231 - 276)	123.4 (115 - 130)	185 (184 - 187)
	7	434 (412 - 470)	181 (180 - 182)	108 (103 - 113)

S5 GAUSSIAN PROCESS REGRESSION AND MODEL VALIDATION

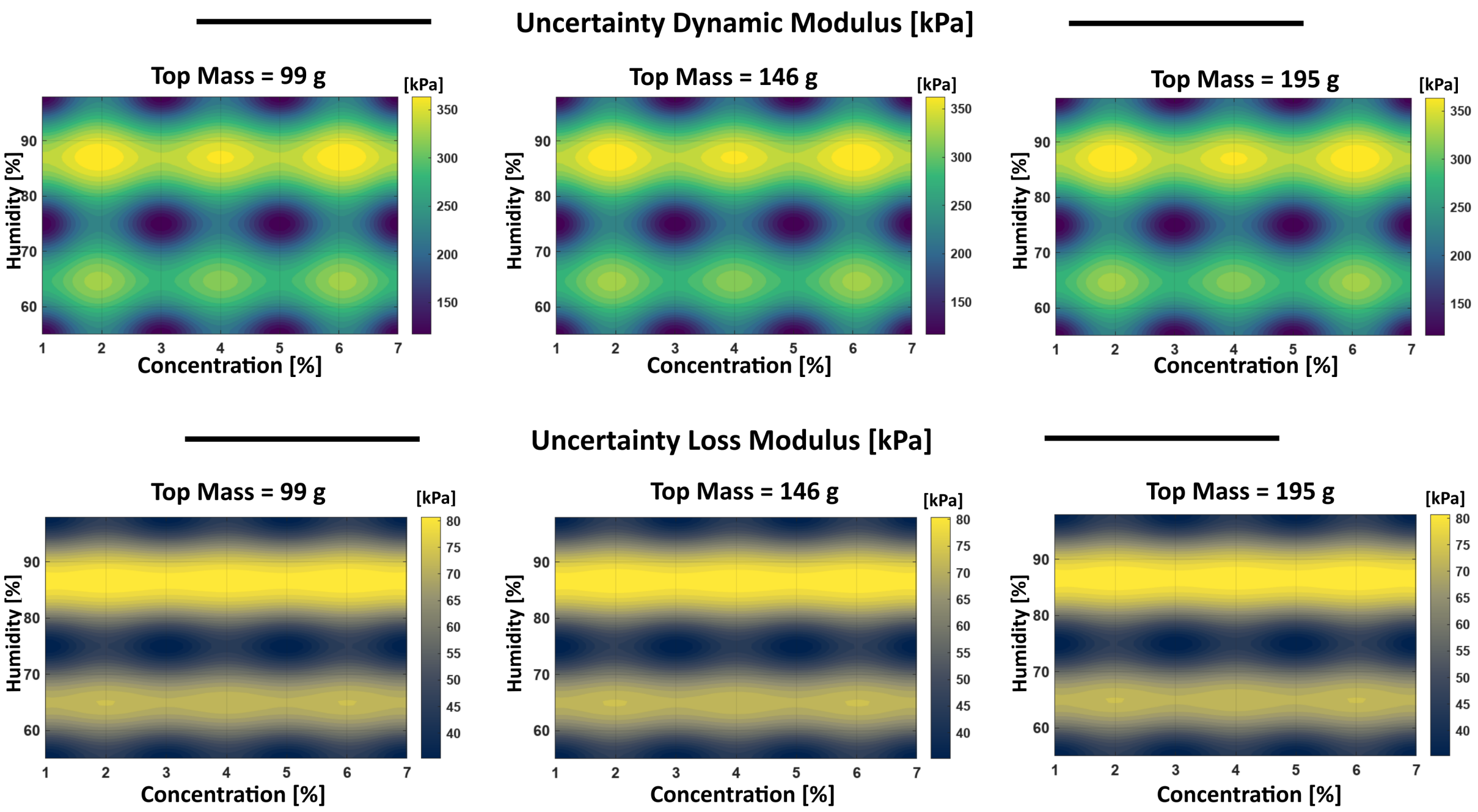

Figure S2: Predictive uncertainty maps of agarose gel viscoelastic properties. The heatmaps display the predictive standard deviation (σ) for the Dynamic Modulus (top row), and Loss Modulus (bottom row) as a function of Agarose Concentration and Relative Humidity. From left to right, the maps illustrate the model uncertainty at increasing pre-stress levels (Top Mass: 99 g, 146 g and 195 g). Darker regions (lower σ) correspond to the high-confidence zones surrounding experimental data points, while lighter regions (higher σ) represent interstitial predictive zones.

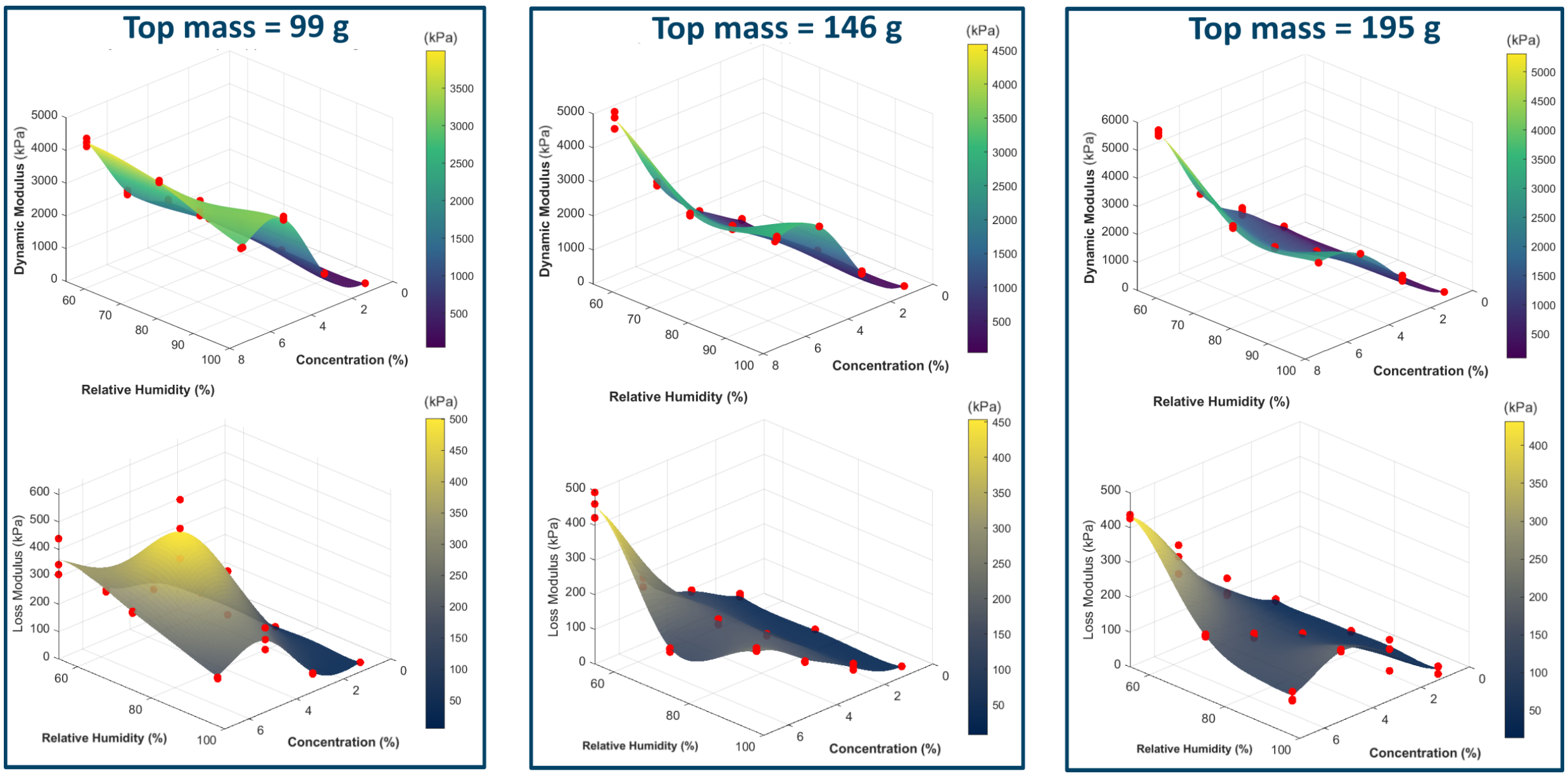

Figure S3: Gaussian Process Regression (GPR) response surfaces of agarose gel viscoelastic properties, experimental data points are overlaid as red spheres.

Table S8: Evaluation of GPR architectures (kernel and basis function combinations) using grouped 3-fold cross-validation for predicting the viscoelastic properties of agarose gels. The table includes the optimized β coefficients.

**Kernel**	**Basis Function**	**CV RMSE**	**CV R^2**	**Beta Coefficients (β)**
		**Dynamic Modulus [kPa]**		
Matérn 3/2	Linear	125	0.99	$[1832.60, 1333.36, 29.28, -128.39]^\top$
Matérn 5/2	Linear	125	0.99	$[1854.86, 1335.53, 30.72, -153.01]^\top$
Sq. Exponential	Linear	125	0.99	$[1868.55, 1343.82, 31.42, -158.30]^\top$
Matérn 3/2	Constant	125	0.99	$[2404.05]^\top$
Matérn 3/2	Pure Quadratic	125	0.99	$[1451.82, 1334.25, 28.39, -144.02, 70.25, 84.69, 172.30]^\top$
Matérn 5/2	Pure Quadratic	125	0.99	$[1467.99, 1338.67, 28.39, -147.99, 67.02, 84.69, 159.21]^\top$
Sq. Exponential	Pure Quadratic	126	0.99	$[1492.71, 1345.42, 28.39, -154.05, 62.07, 84.69, 139.20]^\top$
Matérn 5/2	Constant	126	0.99	$[2211.74]^\top$
Sq. Exponential	Constant	133	0.99	$[1994.31]^\top$
		**Loss Modulus [kPa]**		
Sq. Exponential	Constant	38	0.92	$[152.17]^\top$
Matérn 5/2	Constant	38	0.92	$[154.12]^\top$
Matérn 3/2	Constant	38	0.92	$[155.22]^\top$
Sq. Exponential	Pure Quadratic	39	0.91	$[154.87, 93.00, -18.86, -34.67, -19.71, 16.80, 0.24]^\top$
Sq. Exponential	Linear	39	0.91	$[152.39, 92.56, -18.34, -35.01]^\top$
Matérn 5/2	Pure Quadratic	39	0.91	$[152.26, 93.25, -19.02, -35.04, -18.41, 18.04, 0.41]^\top$
Matérn 3/2	Pure Quadratic	39	0.91	$[150.56, 93.47, -19.20, -35.05, -17.72, 18.53, 0.88]^\top$
Matérn 5/2	Linear	39	0.91	$[152.73, 92.90, -18.44, -35.40]^\top$
Matérn 3/2	Linear	39	0.91	$[152.70, 93.19, -18.59, -35.36]^\top$

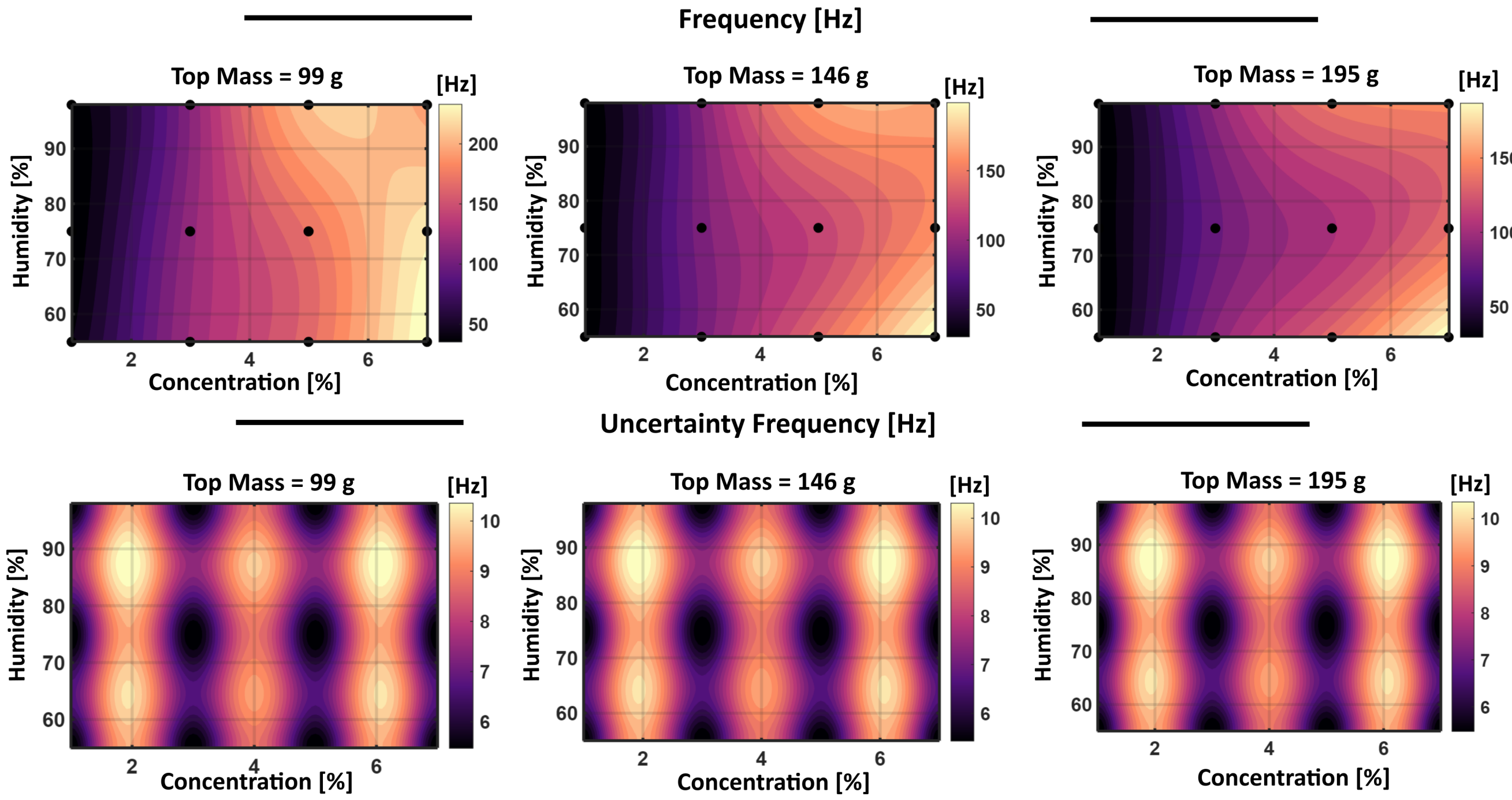

Figure S4: Gaussian Process Regression (GPR) contour maps of predicted resonance frequency (top) and associated model uncertainty (bottom) for agarose hydrogels. The response is evaluated as a function of polymer concentration and relative humidity under increasing compressive preloads (top mass). Black markers indicate the experimental sampling points. The model uses an Automatic Relevance Determination (ARD) `matérn` 5/2 kernel with constant basis set (CV RMSE = 5.88 Hz and $R^2_{\mathrm{CV}} = 0.99$)

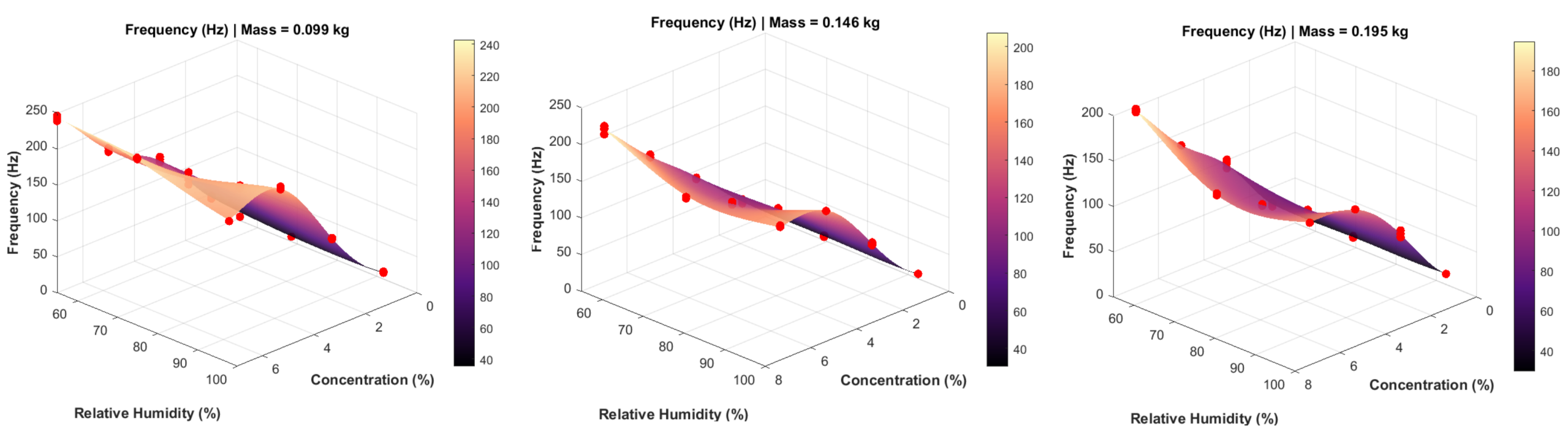

Figure S5: Gaussian Process Regression (GPR) response surfaces illustrating the effect of relative humidity and concentration on the frequency response of agarose gels under increasing preloads. Experimental data points are overlaid as red spheres.

Table S9: Evaluation of GPR architectures (kernel and basis function combinations) using grouped 3-fold cross-validation for predicting the resonance frequency of agarose hydrogels. The optimized β coefficients are reported for each model.

**Kernel**	**Basis Function**	**CV RMSE [Hz]**	**CV R^2**	**Beta Coefficients (β)**
Matérn 3/2	Linear	5.8	0.99	$[128.88, 61.23, -14.17, -5.69]^\top$
Matérn 5/2	Linear	5.8	0.99	$[128.70, 61.08, -14.03, -5.64]^\top$
Matérn 3/2	Constant	5.8	0.99	$[157.34]^\top$
Sq. Exponential	Linear	5.8	0.99	$[126.25, 60.03, -14.41, -4.66]^\top$
Matérn 3/2	Pure Quadratic	5.9	0.99	$[118.12, 60.51, -15.51, -3.34, -12.72, 6.46, 8.25]^\top$
Matérn 5/2	Pure Quadratic	5.9	0.99	$[118.48, 60.39, -15.42, -3.49, -12.80, 6.41, 8.06]^\top$
Sq. Exponential	Pure Quadratic	5.9	0.99	$[119.07, 59.73, -15.36, -3.69, -12.69, 6.25, 7.61]^\top$
Matérn 5/2	Constant	5.9	0.99	$[147.61]^\top$
Sq. Exponential	Constant	6.2	0.99	$[138.09]^\top$

S6 VISCOELASTIC SENSITIVITY MAPS OF AGAROSE HYDROGELS

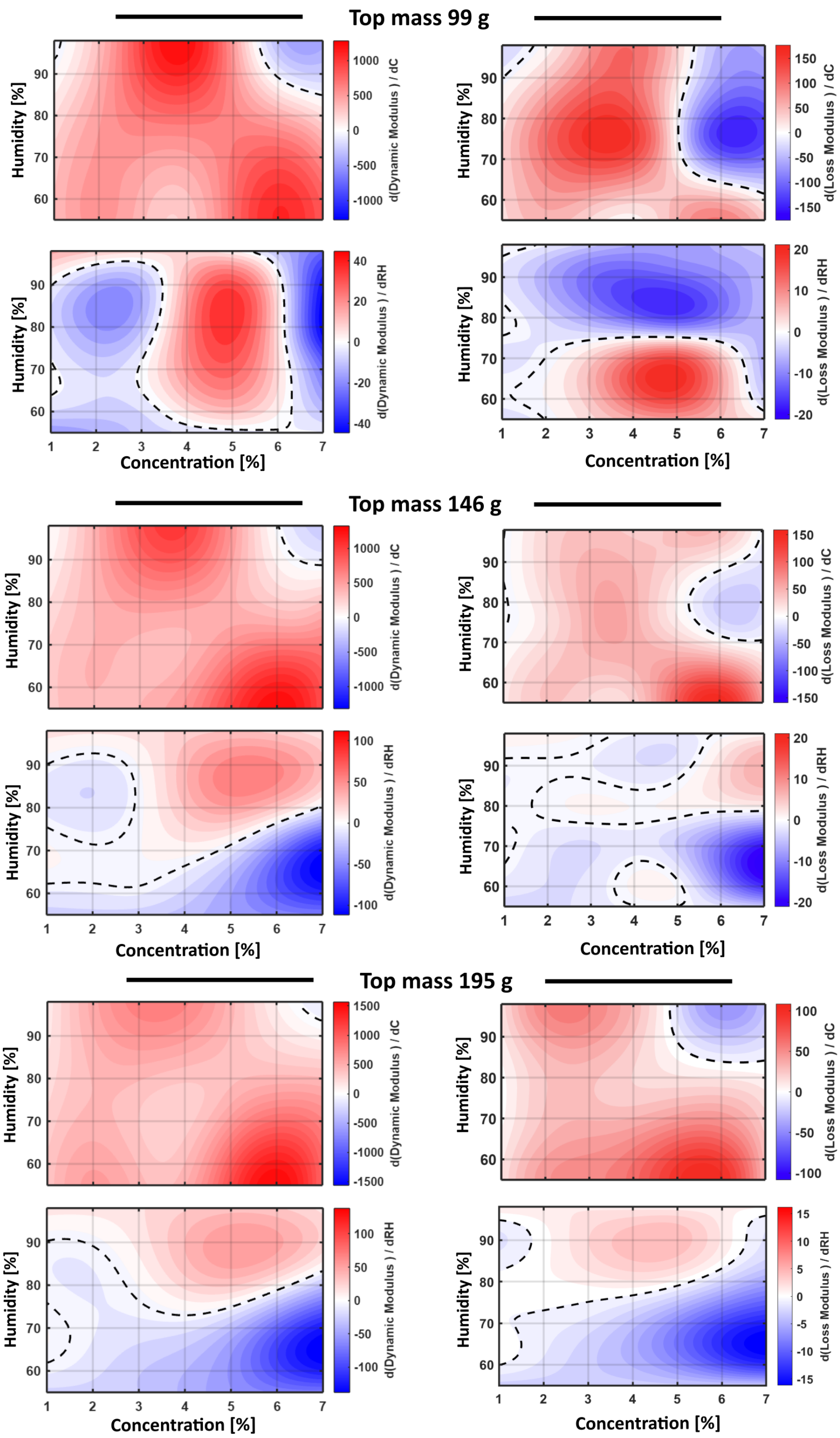

Figure S6: Viscoelastic sensitivity maps of agarose hydrogels evaluated under a different pre-stress mass from 99 g to 195 g. The contour plots visualize the partial derivatives of the material's dynamic response with respect to polymer concentration. Dashed black lines represent zero-gradient contours, delineating critical structural and environmental transition zones where the material properties reach a local maximum, minimum, or plateau.

S7 AGAROSE SAMPLE DATA: DYNAMIC MECHANICAL ANALYSIS

Table S10: Viscoelastic properties measured at a frequency of 28 Hz at 98% of humidity: Storage Modulus (E') and loss modulus (E''). Data are presented as Mean (Min – Max) for $n = 3$.

Conc.	E' [kPa]	E'' [kPa])
1%	32 (26 - 40)	5 (3 - 7)
3%	112 (110 - 114)	13 (12 - 13)
5%	110 (61 - 192)	13 (12 - 13)
7%	251 (170 - 378)	20 (16 - 25)

Table S11: Viscoelastic properties measured at a frequency of 28 Hz at 55% of humidity: Storage Modulus (E') and loss modulus (E''). Data are presented as Mean (Min - Max) for $n = 3$.

Conc.	E' [kPa]	E'' [kPa])
1%	33 (15 - 71)	8 (5 - 14)
3%	117 (82 - 176)	10 (8 - 12)
5%	237 (113 - 368)	20 (14 - 25)
7%	255 (133 - 337)	22 (14 - 27)

S8 QUASI-STATIC COMPRESSION TEST IMAGES

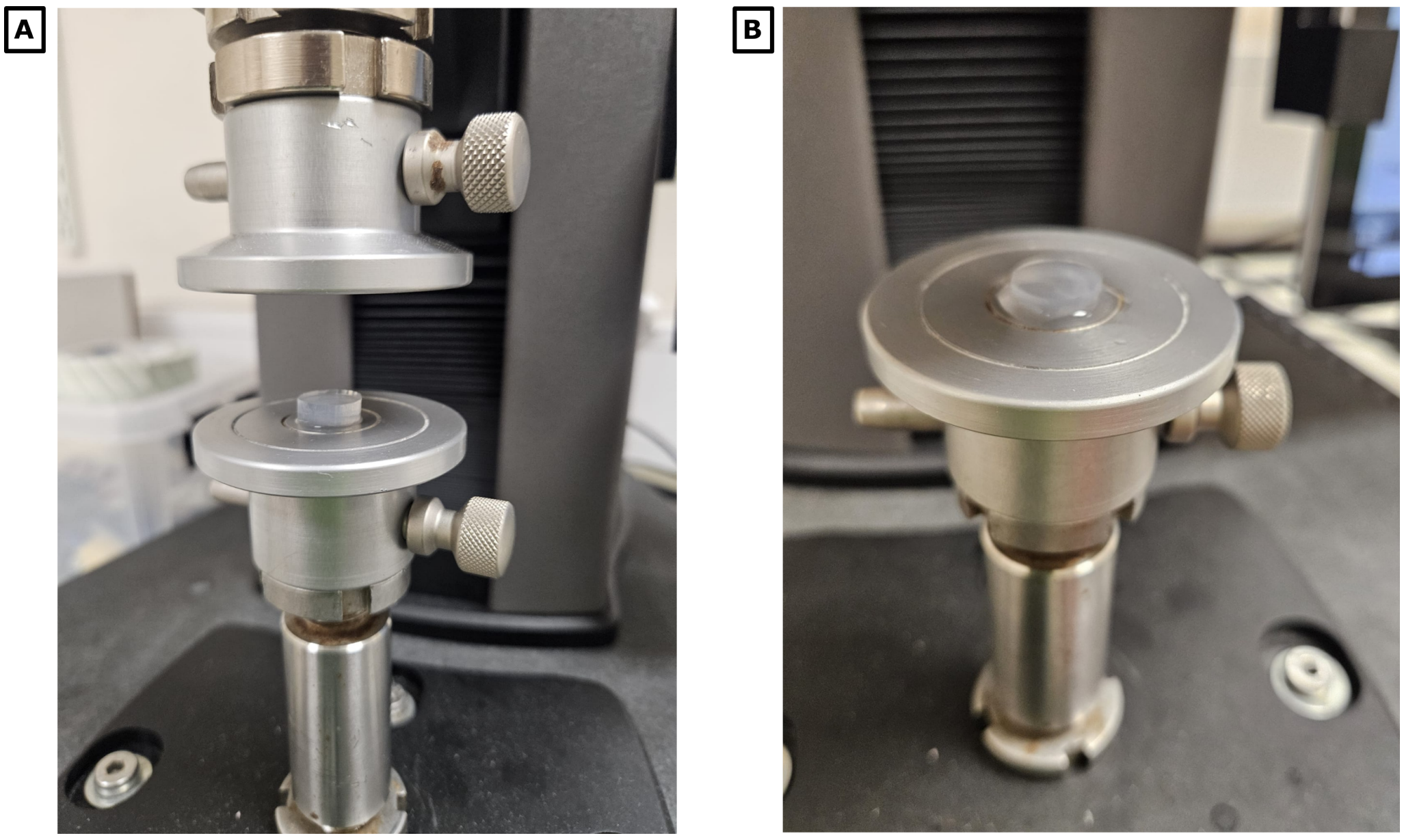

Figure S7: Quasi-static compression teston agarose 1% wt. **A**. sample before the test and **B**. Sample after the test with water exuding from the agarose gel.

S9 VIBRATION TRASMISSIBILITY TEST IMAGES

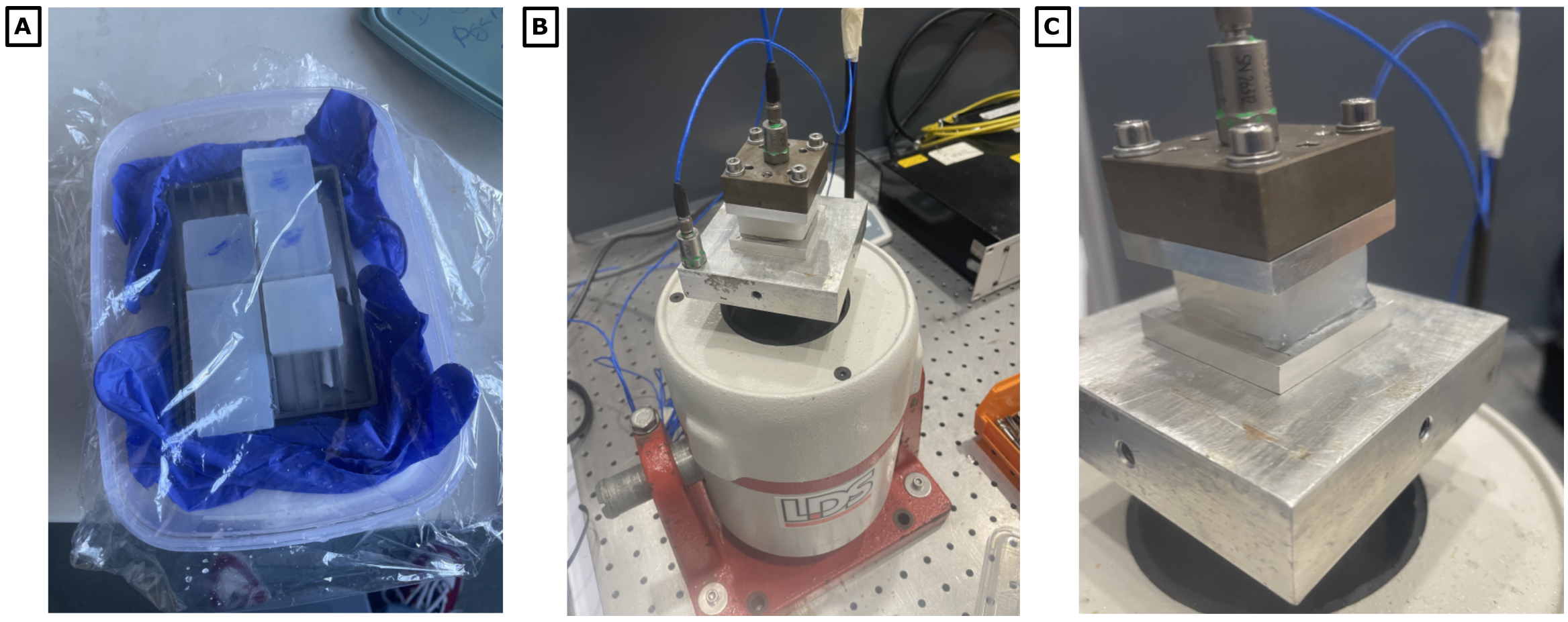

Figure S8: **A**. Air-thight relative humidity conditioning chamber. **B**. Vibration strasmissibility test set-up. **C**. Water exuding from the agarose gel 1% wt after vibration testing.
